# Self-Supporting Hyaluronic Acid-Functionalized G-Quadruplex-Based Perfusable Multicomponent Hydrogels Embedded in Photo-Cross-Linkable Matrices for Bioapplications

**DOI:** 10.1021/acs.biomac.3c00433

**Published:** 2023-06-20

**Authors:** Vera Sousa, Adérito J. R. Amaral, Edgar J. Castanheira, Igor Marques, João M. M. Rodrigues, Vítor Félix, João Borges, João F. Mano

**Affiliations:** CICECO − Aveiro Institute of Materials, Department of Chemistry, University of Aveiro, Campus Universitário de Santiago, 3810-193 Aveiro, Portugal

## Abstract

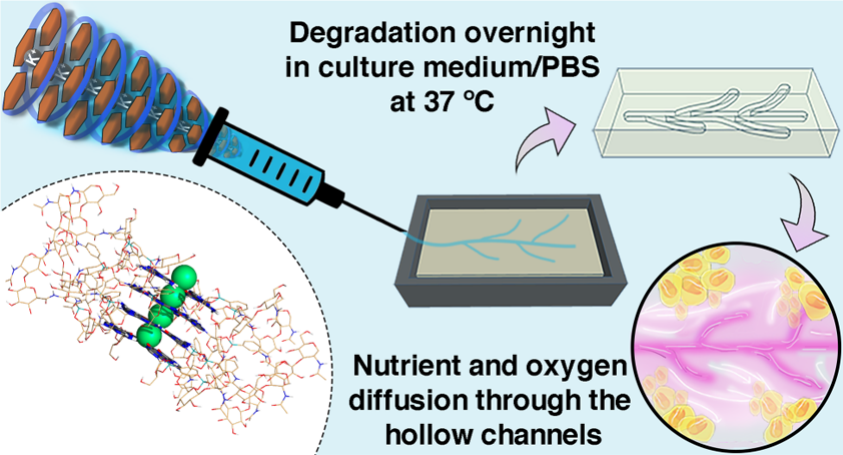

Dynamic G-quadruplex supramolecular hydrogels have aroused great interest in a broad range of bioapplications. However, neither the development of native extracellular matrix (ECM)-derived natural biopolymer-functionalized G-quadruplex hydrogels nor their use to create perfusable self-supporting hydrogels has been explored to date, despite their intrinsic potential as carrier vehicles of therapeutic agents, or even living cells in advanced regenerative therapies, or as platforms to enable the diffusion of nutrients and oxygen to sustain long-term cell survival. Herein, we developed a dynamic co-assembling multicomponent system that integrates guanosine (G), 3-aminophenylboronic acid functionalized hyaluronic acid (HA-PBA), and potassium chloride to bioengineer strong, homogeneous, and transparent HA-functionalized G-quadruplex hydrogels with injectable, thermo-reversible, conductive, and self-healing properties. The supramolecular polymeric hydrogels were developed by hydrogen bonding and *π–π* stacking interactions between G coupled *via* dynamic covalent boronate ester bonds to HA-PBA and stabilized by K^+^ ions, as demonstrated by a combination of experiments and molecular dynamics simulations. The intrinsic instability of the self-assembled G-quadruplex structures was used to bioengineer self-supporting perfusable multicomponent hydrogels with interconnected size and shape-tunable hollow microchannels when embedded in 3D methacrylated gelatin supporting matrices. The microchannel-embedded 3D constructs have shown enhanced cell viability when compared to the bulk hydrogels, holding great promise for being use as artificial vessels for enabling the diffusion of nutrients and oxygen essential for cell survival. The proposed approach opens new avenues on the use of ECM-derived natural biopolymer-functionalized dynamic G-quadruplex hydrogels to design next-generation smart systems for being used in tissue regeneration, drug screening, or organ-on-a-chip.

## Introduction

Nature provides us with an unparalleled array of stunning supramolecular landscapes formed through the molecular self-assembly of nucleobases, peptides, phospholipids, saccharides, or their analogues. Such small yet powerful fundamental building blocks of life work as an unprecedented source of inspiration for engineering a library of complex and biofunctional supramolecular architectures for bioapplications.^1^ In particular, G-quadruplexes are ubiquitous noncanonical four-stranded supramolecular structures formed in guanine-rich DNA and RNA sequences within cells that have arisen considerable interest owing to their unique self-assembled structures and multiple biological roles.^2−7^ The G-quadruplex structural stability can be influenced by the direction of the G-strands, length, position, and composition of the connecting loops.^8^ Furthermore, the length of the G-strands and the guanine conformation dictate the arrangement of the G-quadruplex structure, which can either be parallel, antiparallel, or hybrid.^9^

The building blocks of supramolecular G-quadruplexes are G-quartets (G4), which are formed by the supramolecular Hoogsteen-type hydrogen bonding interactions between either four guanine units or their derivatives in a square planar arrangement and stabilized *via* metal coordination by resorting to alkali metal cations, such as K^+^, Na^+^, or NH_4_^+^.^10−12^ Furthermore, higher-order G-quadruplex supramolecular structures are obtained by the π–π stacking interactions of G4 monomers, leading to a columnar self-assembled four-stranded helical structure denoting dynamic features.^2,13−15^ These columnar aggregates can retain a large amount of water and be extended to generate an entangled self-assembled nanofibrillar network that leads to a supramolecular hydrogel.^16,17^ In addition, the biocompatibility and biodegradability entailed by the G4 self-assembled structures turn them into very appealing supramolecular bioarchitectures for the development of polymeric functional supramolecular hydrogels for bioapplications.^16^ Those include applications in sustained drug/therapeutics delivery,^18−20^ cell culture,^21^ tissue engineering,^22^ sensing and imaging,^23^ or medical diagnosis.^24^

Frequently, guanosine (G) and its analogues are conjugated with low- and high-molecular-weight molecules to enhance the self-assembly driven hydrogelation, increase the lifetime stability, and assign dynamic multifunctional properties to the developed G-quadruplex supramolecular structures.^25−28^ In particular, boronic acid is a highly versatile and one of the most commonly used building blocks for multicomponent self-assembly due to its ability to bind molecules containing diol groups, namely *cis*-1,2-diols,^16,18,25,29−38^ such as G. Thereby, it enables the formation of dynamic and reversible cyclic boronate ester bonds-mediated G-quadruplex hydrogels denoting stimuli-responsive behavior, self-healing, injectable, or shape memory properties.^18,25,29,38−42^ Recently, Biswas et al. engineered a dynamic, stimuli-sensitive polyethylene (PEG)-functionalized G-quadruplex hydrogel for sustained doxorubicin release using 2-formylphenylboronic acid (2-FPBA) and G.^18^ Moreover, Tanaka et al. reported the preparation of biodegradable and self-healable supramolecular G-quadruplex hydrogels by directly and symmetrically coupling deoxyguanosine monomers to the ends of the PEG chains, in the presence of K^+^ or Na^+^, holding great promise to be used as drug carriers.^43^ Besides their intrinsic potential for biomedical applications, G-quadruplex hydrogels can find widespread applications. For instance, G-quadruplex hydrogels formed *via* hydrogen bonding interactions between the phosphate group on guanosine monophosphate and the -NH_2_ group on poly(ethylenimine) were found to be efficient contaminant adsorbents.^44^ However, the G-quadruplex hydrogels developed to date remain mostly unstable over time and disassemble mainly due to the propensity of G to precipitate and the need for a high concentration of alkali metal cation, which imposes a major burden for bioapplications.^45^ Moreover, their hydrophilic nature also hinders the G-quadruplex stability,^46,47^ which could be used as a source of inspiration to develop perfusable supramolecular biomaterials for a wide array of bioapplications. To date, several micro- and nanofabrication techniques, including the layer-by-layer (LbL) assembly technology, sacrificial molding, and bioprinting have been widely used to generate perfusable channels in hydrogel scaffolds.^48−52^ However, those technologies entail major bottlenecks. For instance, the commonly employed dip-assisted LbL technology is time-consuming and requires a large amount of material, while the sacrificial molding techniques rely on the use of cytotoxic organic solvents or harmful conditions to remove the sacrificial structures, being laborious and difficult to scale-up. On the other hand, the bioprinting technologies entail high costs and limitations on the printing resolution and speed and on the type of biomaterials and cells that can be printed.^53^

Herein, we explored the well-known instability of the G-quadruplex hydrogels to produce a self-degradable multicomponent biomaterial in an easy, fast, cost-effective, and reproducible way. This work reports for the first time the use of hyaluronic acid (HA), a biocompatible, biodegradable, and ubiquitous natural biopolymer in the native extracellular matrices (ECM) of tissues, and its functionalization with boronic acid moieties to bioengineer dynamic HA-functionalized G-quadruplex based supramolecular hydrogels by multicomponent self-assembly of G and 3-aminophenylboronic acid functionalized HA (HA-PBA) in the presence of KCl. The G-quadruplex hydrogel was further embedded in a biocompatible and biodegradable gelatin methacrylated (GelMA) prehydrogel supporting matrix to develop robust photo-cross-linkable 3D constructs for biomedical applications. The simple exposure of the construct to culture media conducted to the on-demand self-degradation and, thus, disassembly of the G-quadruplex structure at physiological conditions, without the need for any external chemical and/or physical stimuli, generating perfusable microchannels within the GelMA supporting matrix. The inherent versatility imparted by the proposed approach enables the fabrication of cytocompatible, customizable and perfusable supramolecular structures embedded in virtually any kind of photo-cross-linkable hydrogels, opening new avenues in controlled therapeutics/cells delivery, modular tissue engineering, and regenerative medicine strategies.

## Experimental Section

### Materials

Guanosine (G), 3-aminophenylboronic acid hydrochloride (PBA) and gelatin (Gel) were purchased from Sigma-Aldrich. Low-molecular-weight hyaluronic acid sodium salt (*M*_W_ = 80–100 kDa) and potassium chloride (KCl) were purchased from Biosynth Carbosynth and ChemLab, respectively. The 4-(4,6-dimethoxy-1,3,5-triazin-2-yl)-4-methylmorpholinium chloride (DMTMM) and lithium phenyl(2,4,6-trimethylbenzoyl)phosphinate (LAP) were bought from TCI Chemicals and Carbosynth, respectively. The 2-(*N*-morpholino)ethanesulfonic acid buffer (MES, 0.1 M, pH 6.0) and Trizma buffer (68 mM, pH 7.4) were acquired from Alfa Aesar and Sigma-Aldrich, respectively, and prepared in ultrapure water from a Milli-Q Plus water purification system (resistivity > 18.2 MΩ·cm) from Millipore. Unless otherwise specified, all chemicals were used as received without further purification.

### Chemical Functionalization of Hyaluronic Acid with 3-Aminophenylboronic Acid

HA-PBA polymer was prepared by conjugating PBA to HA, using DMTMM as the coupling agent.^54^ Briefly, 1 g of HA (2.492 mmol) was completely dissolved in 90 mL of MES (0.1 M, pH 6) under constant stirring. Next, DMTMM (689.6 mg, 2.492 mmol) was separately dissolved in 5 mL of MES, added to HA solution and stirred for 30 min to activate the carboxyl groups of HA. Then, PBA (432.1 mg, 2.492 mmol) was dissolved in 5 mL of MES and finally added to the HA solution. The reaction mixture was stirred at room temperature (RT) in N_2_ atmosphere for 72 h. Afterward, the reaction mixture was purified by dialysis against distilled water using a membrane with a molecular weight cut-off (MWCO) of 3.5 kDa. The purified HA-PBA was obtained by freeze-drying, giving rise to a white foam. The degree of substitution (DS) was calculated by proton nuclear magnetic resonance (^1^H NMR) spectroscopy, as reported elsewhere.^54^^1^H NMR (300.13 MHz, D_2_O): δ (ppm) 1.99 (s, 3H, −CH_3_), 2.90–4.72 (HA backbone), 7.45–7.87 (m, 4H, −C_6_H_4_).

### Preparation of the HA-Functionalized G-Quadruplex Hydrogels

For the preparation of the HA-functionalized G-quadruplex hydrogels, 1.5, 2.5, and 3.5% (w/v) of HA-PBA and 42, 70, and 98 mM of G, respectively, were added to 1 mL of Trizma buffer solution (68 mM, pH = 7.4) and mechanically stirred at physiological pH in a glass vial. Then, the precursor mixture was heated at 80 °C until the solution became clear. At this point, 250 mM of KCl were added. The resulting mixture was then cooled down to RT and the gelation time assessed by the vial inversion test, which was completed after 2 min, giving rise to a transparent and self-supporting gel.

### Metal Ion Affinity Study

The formation, stability, and affinity of the HA-functionalized G-quadruplex hydrogels to different metal cations were assessed by preparing the hydrogel at 1.5% (w/v) using MgCl_2_, CaCl_2_, NH_4_Cl, NaCl, LiCl, CsCl, or KCl at 250 mM.

### Molecular Modeling

Molecular dynamics (MD) simulations were carried out with the Amber20 software suite, under periodic boundary conditions in water solution. The HA-functionalized G-quadruplex model was described with the restrained electrostatic potential (RESP) atomic charges and force field parameters taken from the general AMBER force field 2 (GAFF2), except for boron. The bonding terms and van der Waals parameters involving this atomic center were derivatized as detailed in the Supporting Information (SI). The monatomic anions were described with van der Waals parameters developed for the transferable intermolecular potential with 3 points (TIP3P) water model, while NH_4_^+^ was described with GAFF2 parameters and RESP charges. The MD simulations were carried out following a multistage protocol, with sampling data collected in an isothermal–isobaric (NPT) ensemble, as described below.

### Proton Nuclear Magnetic Resonance (^1^H NMR) Spectroscopy

The ^1^H NMR spectra of the HA-functionalized G-quadruplex hydrogel, HA-PBA, and G were recorded on an Avance II 300 MHz spectrometer (Bruker, Germany) at 300.13 MHz using tetramethylsilane (TMS) as internal reference. To perform the ^1^H NMR spectrum of the HA-functionalized G-quadruplex hydrogel, the desired amount of G and HA-PBA were dissolved in 468.8 μL of D_2_O and heated to 80 °C. The resulting clear solution was then transferred into an NMR tube and 31.2 μL of freshly prepared 4 M KCl solution (*C*_f_ = 250 mM) was added to enable the formation of the gel. To study the influence of the solution pH on the formation of boronate ester bonds, the ^1^H NMR spectra was acquired under acidic (pH 4) and basic conditions (pH 10).

### Attenuated Total Reflectance-Fourier Transform Infrared (ATR-FTIR) Spectroscopy

A Tensor 27 FTIR spectrometer (Bruker, Germany) fitted with a “Golden Gate” ATR module equipped with a diamond crystal was used to collect the spectra of the lyophilized G-quadruplex hydrogel (1.5%, w/v), HA-PBA, and G in the transmittance mode. All data were obtained in the spectral range of 4000–400 cm^–1^ by averaging 256 individual scans per sample at a resolution of 4 cm^–1^.

### Wide-Angle Powder X-ray Diffraction (PXRD)

The wide-angle PXRD spectra of the freeze-dried G-quadruplex hydrogel at 1.5% (w/v), HA-PBA and G were collected at 25 °C on an Empyrean PANalytical diffractometer (Malvern PANalytical, U.K.) equipped with a PIXcel1D detector (active length = 3.3473°) and a spinner flat sample holder in a Bragg–Brentano para-focusing optics configuration. Diffraction intensity data were collected in the 2θ range between 1° and 40° using CuKα radiation filtered in Ni under beam conditions of 45 kV and 40 mA. Data were collected by the continuous counting method assuming a scanning step size of 0.0263° and a scanning rate of 0.02° min^–1^. The wide-angle PXRD patterns were analyzed with the HighScore Plus software and ICDD PDF4+ (2019 release) database. The stacking distance (*d*) was calculated according to the Bragg’s law.^55^

### Thioflavin T (ThT) Fluorescence Assay

One mL of ThT (1 mM) loaded HA-functionalized G-quadruplex hydrogel, at the highest (3.5%) and lowest (1.5%) concentrations, was transferred to a quartz cuvette. The spectra were recorded on a FP-8300 fluorometer (JASCO, Japan) at 25 °C, the bandwidth for the emission spectra was set at 2.5 nm, and the data pitch at 1 nm. The spectra were obtained at λ_ex_ of 450 nm in a data range of 460–750 nm. Also, the spectra of the ThT solution and the precursor mixture without K^+^ were recorded for comparison.

### Circular Dichroism (CD)

The CD spectrum of the diluted and freshly prepared G-quadruplex hydrogel (250 μg mL^-1^) was recorded at 25 °C on a J-1500 CD spectrometer (JASCO, Japan) from 190 to 350 nm at a scan rate of 100 nm min^–1^ with a data pitch of 1.0 nm. The CD spectrum was obtained as the average of three accumulations.

### Scanning Transmission Electron Microscopy (STEM)

STEM analysis of the G-quadruplex hydrogel, diluted to 500 μg mL^-1^, was performed on a field emission gun scanning transmission electron microscope (Hitachi STEM HD-2700, Hitachi High-Technologies, Japan) operated at an acceleration voltage of 200 kV. Prior to the STEM analysis, a 5 μL of the diluted hydrogel was drop casted onto carbon film-coated copper TEM grids (CF400-Cu-carbon film 400 square mesh copper grid) and the excess was removed by micropipette. Afterward, the grids were air-dried in the fume hood overnight at RT.

### Rheology Measurements

A Kinexus Lab+ rotational rheometer (Malvern PANalytical, U.K.) equipped with a stainless-steel parallel plate geometry (8.0 mm diameter) and a solvent trap was used to characterize the rheological properties of the hydrogels at all tested concentrations. The gap setting was fixed at 1.0 mm, the tests were performed at 25 °C with a frequency of 1.0 Hz and the shear strain was fixed within the linear viscoelastic region (LVR). All data were analyzed in triplicates. Oscillatory strain amplitude sweep measurements at a frequency of 1 Hz were initially carried out to determine the LVR. To evaluate the viscoelastic behavior of the hydrogels, oscillatory frequency sweep tests were performed at a constant shear strain of 0.5% to measure the storage (*G*′) and loss (*G*″) moduli. Temperature sweep measurements were performed to study the storage moduli behavior within the range of 80–25 °C. The shear-thinning profile of the hydrogels was assessed by resorting to the analysis of the shear viscosity (Pa·s) while the shear rate (s^–1^) increased. Lastly, the self-healing ability of the hydrogels was also assessed by dynamic rheology by employing step strain experiments. First, the hydrogels were cut through the center into two pieces and rejoined into one integral piece at 25 °C. Then, the recovery of their storage moduli was studied. In this experiment, alternate low (0.1%) and high (50/100%) strains were applied over ten cycles and the self-healing efficiency was calculated for the first and last deformation cycle as the ratio between the *G*′ value of the healed gel and the original *G*′ value. In addition, the viscoelastic behavior of the GelMA 10% (w/v) precursor solution during the photopolymerization process was analyzed by photorheology through irradiation with blue light (λ = 385–515 nm, ValoTM grand, 1000 mW/cm^2^) at 25 °C within the LVR (0.03% strain).

### Water Content

After the preparation of the 1.5, 2.5, and 3.5% (w/v) HA-functionalized G-quadruplex hydrogels, the excess of water was removed, and the wet weight (*W*_w_) measured. Then, the hydrogels were frozen at −80 °C, freeze-dried for 24 h, and the dry weight (*D*_w_) was measured. The water content was calculated as shown in the eq 1:
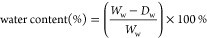
1

### Injectability Test

The G-quadruplex hydrogels at 1.5, 2.5, and 3.5% (w/v) were loaded each into a 1 mL syringe and injected through needles with either 25, 21, or 16 G corresponding to inner diameters of 260 μm, 514 μm, or 1.194 mm, respectively, to obtain needle-shaped fibers.

### Self-Healing and Conductivity Tests

The self-healing ability of the HA-functionalized G-quadruplex hydrogels was also visually studied. First, the hydrogel was cut in half through the center and the two pieces stained with two different dyes. Then, the two pieces were rejoined for 30 min at 25 °C. The healed hydrogel was subjected to a gravity-defying test and stirred in water at 100 rpm to prove the self-healing properties. A conductivity test was performed using two G-quadruplex hydrogels wired to a battery-powered circuit with a LED indicator.

### Fabrication of 3D Constructs Embedding Perfusable Microchannels

After cooling to RT and consequent hydrogelation, the G-quadruplex hydrogel was loaded into a 1 mL syringe and extruded through a needle with either 21 or 16 G. To engineer the 3D constructs embedding the perfusable microchannels, different methacrylated polymer baths were used (methacrylated gelatin, GelMA 10%; methacrylated laminarin, LamMA 5%; methacrylated dextran, DexMA 5%; and methacrylated hyaluronic acid, HAMA 5%). Briefly, 200 μL of each methacrylated biopolymer solution was first added into poly(dimethylsiloxane) (PDMS) molds followed by a rapid (10 s) pre-photopolymerization of the polymer bath using LAP (0.5%) as photoinitiator. Afterward, the HA-functionalized G-quadruplex fibers were placed in the desired pattern and another 200 μL aliquot of the precursor solution was transferred to the mold to complete the final volume of the 3D construct. Finally, the full 3D construct embedding the perfusable microchannel was photopolymerized through irradiation with blue light (λ = 385–515 nm, Valo TM grand, 1000 mW/cm^2^) for 5 min.

### Scanning Electron Microscopy (SEM)

The morphology of the hollow microchannels and their interconnection were analyzed in a field emission gun scanning electron microscope (FESEM Hitachi SU-3800, Japan) coupled with a Bruker Quantax compact 30 detector operated in the secondary electrons mode at an acceleration voltage of 15 kV. Prior to the analysis, the samples were dried with a gradient of ethanol (10, 30, 50, 80, and 100%, for 30 min in each solution), dehydrated overnight at RT, fixed on aluminum stubs by double-sided carbon conductive adhesive tape, and sputtered coated with carbon (K950X Turbo Evaporator, Emitech, U.S.).

### Cell Culture

Human adipose stem cells (ATCC PCS-500-011; hASCs) were seeded in tissue culture flasks T-175 (Sarstedt) using α-MEM (minimum essential medium, ThermoFisher Scientific, supplemented with 10% FBS and 1% antibiotic/antimycotic) under controlled atmosphere of 5% CO_2_ at 37 °C. Cells were trypsinized, soon after reaching confluence and encapsulated in each hydrogel (L × W × H, 10 × 8 × 5 mm) with a density of 2 × 10^6^ cells per mL, on 6-well plates for 3, 5, and 7 days of culture.

### Live/Dead Assay

The 3D constructs were incubated with 2 μL of calcein-AM solution at 4 × 10^–3^ M in DMSO and 1 μL of propidium iodide (PI) at 1 mg/mL (live/dead Kit, ThermoFischer Scientific) in a final volume of 1 mL of PBS at 37 °C for 30 min. After washing with PBS, the constructs were imaged using a Zeiss Axio Imager M2 upright fluorescence microscope (Carl, Zeiss, Germany). Final data was acquired and processed in the Zeiss ZEN v2.3 blue edition software. Additionally, the quantification of the cell viability was assessed by measuring the fluorescence intensity using ImageJ software (Fiji 1.52n).

### Statistical Analysis

The data are presented as mean ± standard deviation (SD) of three independent experiments (*n* = 3) and analyzed using the GraphPad Prism version 8.0.1 (GraphPad software, U.S.A.). Statistically significant differences were evaluated by unpaired *t* test and the level of significance was set at probabilities of *p < 0.05.

## Results and Discussion

### Formation and Stability of G-Quadruplex Supramolecular Hydrogels

#### Experimental Data

**Scheme 1:**
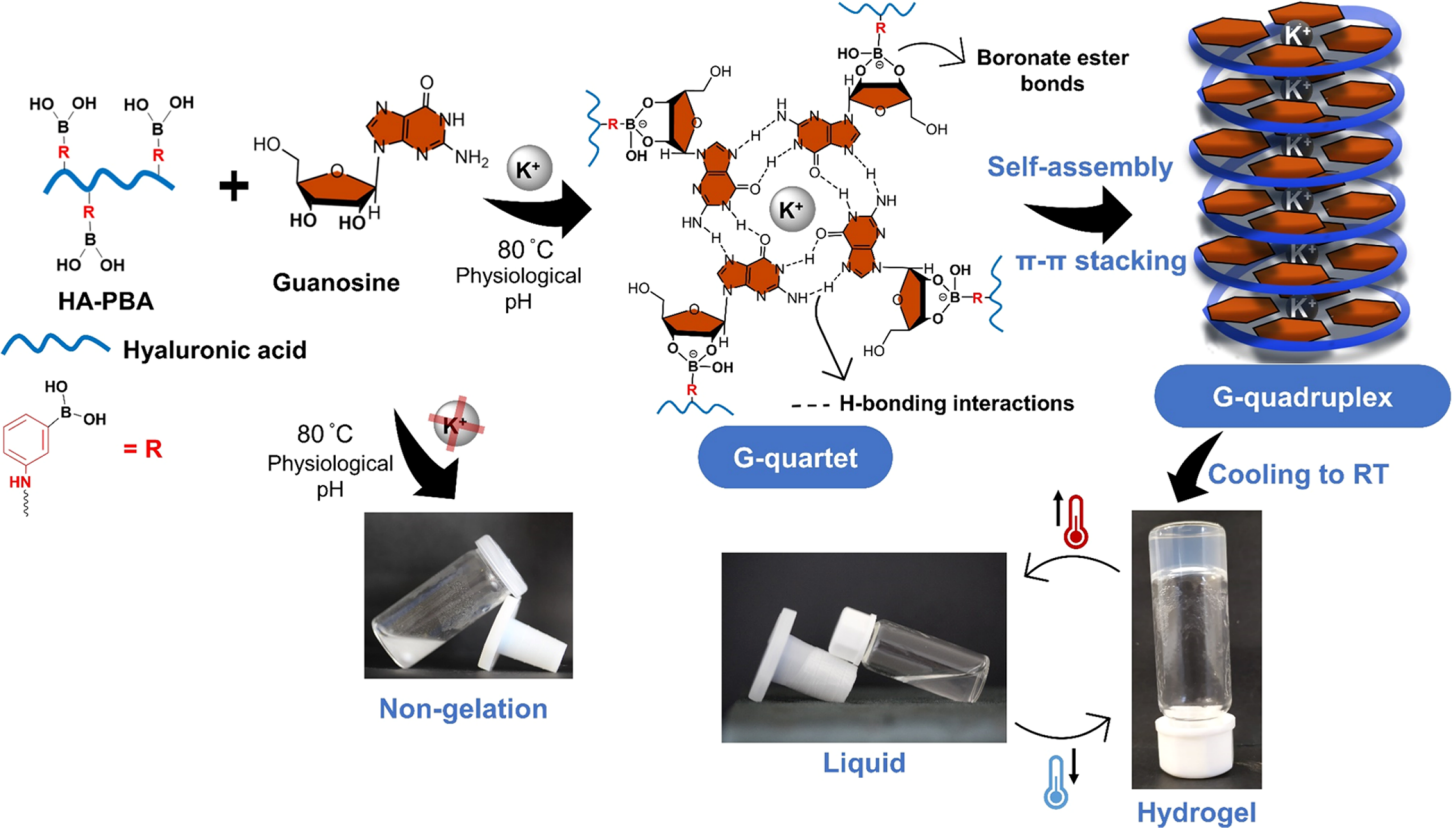
Schematic Illustration of the Mechanism Behind the Formation of HA-Functionalized G-Quadruplex Self-Assembled Hydrogel, with a Thermoresponsive Character, By Combining HA-PBA with G in the Presence of KCl at pH 7.4; Additionally, in a Mixture Lacking K^+^ Ions, Hydrogelation Was Not Achieved

**Figure 1:**
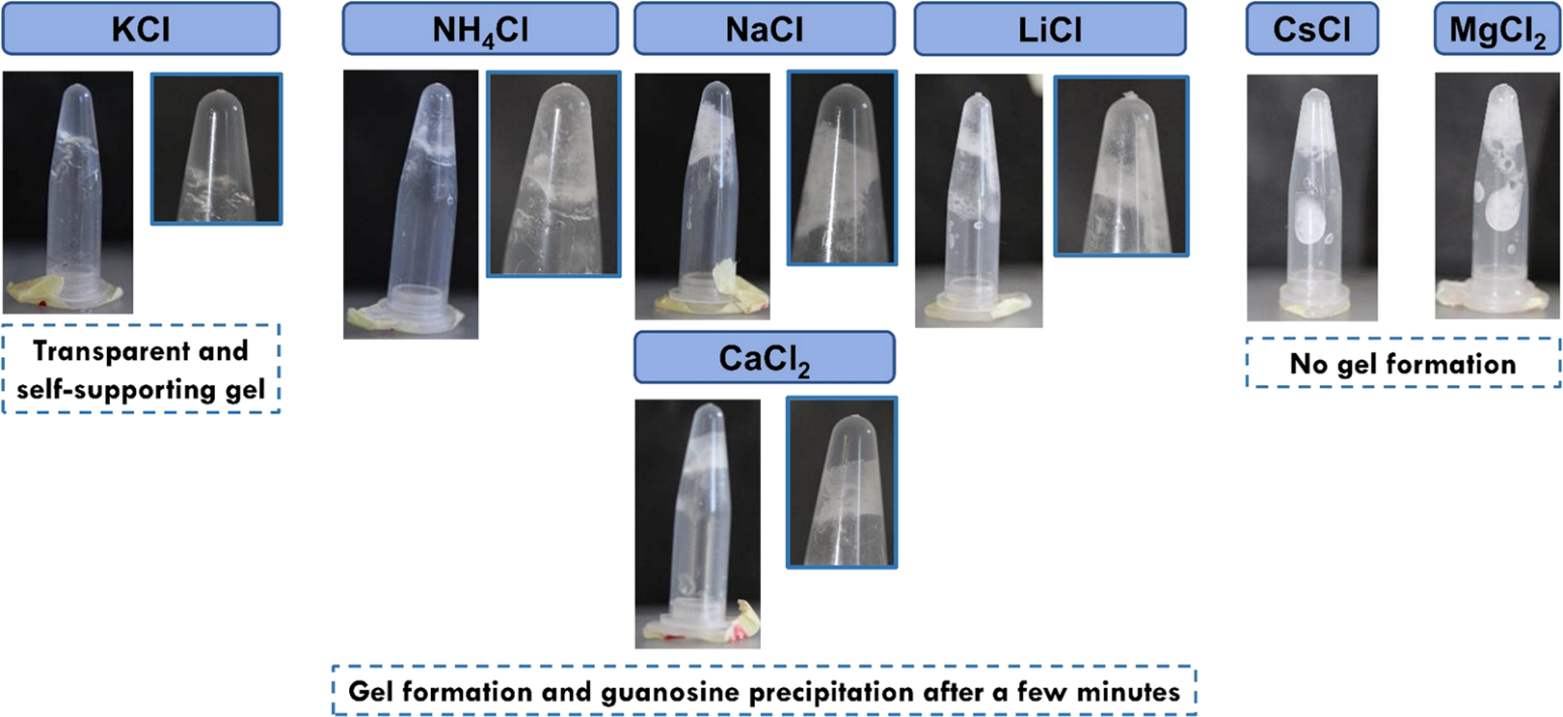
Optical photographs of the alkali metal cation-induced HA-functionalized G-quadruplex hydrogels (1.5% w/v) formation at pH 7.4 assessed *via*the vial inversion method. The selectivity of the self-assembled system was assessed for: KCl, NH_4_Cl, NaCl, CaCl_2_, LiCl, CsCl, and MgCl_2_ at 250 mM.

In this work, a novel HA-functionalized G-quadruplex supramolecular hydrogel was assembled by the π–π stacking of hydrogen-bonded G4 monomers coupled *via* dynamic and reversible cyclic boronate ester bonds (B–O–C) to HA-PBA and stabilized by K^+^ ions (Scheme 1). The functionalization of low-molecular-weight HA (*M*_w_ = 80–100 kDa) with PBA moieties was attempted as described elsewhere (Figure S1a, SI).^54^ The chemical modification of HA was confirmed by ^1^H NMR, denoting a DS of 25.5% (Figure S1b, SI). Then, strong and self-standing transparent HA-functionalized G-quadruplex supramolecular hydrogels were prepared at 1.5, 2.5, and 3.5% (w/v), *via* multicomponent self-assembly, by heating at 80 °C a water-insoluble G solution containing HA-PBA and KCl at a specific molar ratio (Table S1, SI), followed by cooling at RT (Scheme 1). In addition, we assessed the potential formation and stability of the G-quadruplex self-assembled hydrogels by resorting to other monovalent (Na^+^, Li^+^, NH_4_^+^, and Cs^+^) cations and even divalent (Mg^2+^ and Ca^2+^) alkaline earth metal cations, aiming to elucidate the impact of the ionic radius on the HA-functionalized G-quadruplex hydrogelation ability (Figure 1). In opposition to what was found for the K^+^ (1.52 Å)^32^ stabilized G-quadruplex supramolecular hydrogel in which no sign of crystallization of G was observed, the metal ions Cs^+^ (1.81 Å)^32^ and Mg^2+^ (0.81 Å)^56^ did not support hydrogelation. Moreover, although G-quadruplex hydrogels were formed when resorting to Li^+^ (0.90 Å),^32^ Na^+^ (1.16 Å),^32^ Ca^2+^ (1.06 Å),^56^ and NH_4_^+^ (1.43 Å),^57^ the precipitation of G within the hydrogel matrix occurred after a few minutes, leading to the collapse of the loosely formed supramolecular hydrogels.

As such, for the range of tested metal cations, those having ionic radii in the range 0.90–1.52 Å enabled the formation of G-quadruplex supramolecular hydrogels, although stable and self-supporting transparent hydrogels, without any traces of precipitated G, were just formed in the presence of K^+^ ions. Such behavior reveals the high selectivity of the G4 cavities toward this metal cation. On the other hand, the sizes of Mg^2+^ and Cs^+^ are too small and too large to fit inside the G4 cavities, respectively, thus do not enabling the formation of a gel. Therefore, the experimental results reveal the selectivity and lifetime stability of the G-quadruplex hydrogel toward different metal ions, following the trend K^+^ > NH_4_^+^ > Na^+^ = Ca^2+^ > Li^+^ > Cs^+^ = Mg^2+^.

#### MD Simulations

MD simulations were carried out in water to obtain further structural insights into the stability and selectivity of the G4 cavities toward the different alkali metal cations aiming to assemble the HA-functionalized G-quadruplex supramolecular hydrogels.^58−60^ Inspired by a DNA-based parallel G-quadruplex (e.g., PDB 2O4F),^61^ the computational studies were performed using a simplified model composed of four π–π stacked G4s assembled in a G-quadruplex fashion.

**Figure 2:**
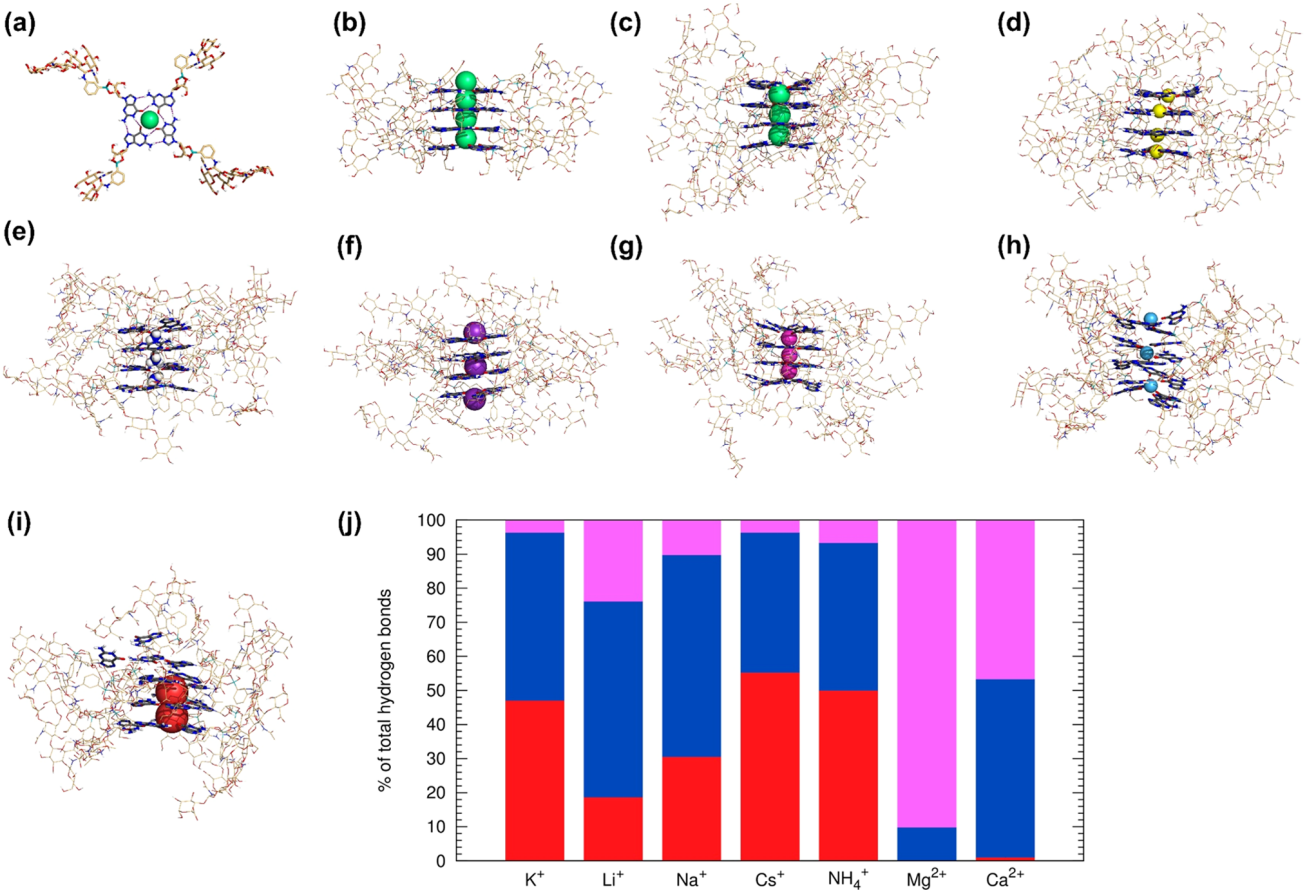
Gas-phase molecular mechanics minimized structures of a (a) G4 or (b) HA-functionalized G-quadruplex model, coordinated to 1 or 4 K^+^ cations (green spheres), in top and side views (see also Figure S2 in the SI), together with representative MD simulations’ snapshots of the model G-quadruplex associations with (c) K^+^; (d) Li^+^ (yellow spheres); (e) NH_4_^+^ (in spheres fashion); (f) Ca^2+^ (purple spheres); (g) Na^+^ (magenta spheres); (h) Mg^2+^ (light blue spheres); and (i) Cs^+^ (dark red spheres). Boron is colored in teal, carbon is in light orange or gray (guanine bases), and the remaining elements follow the typical CPK coloring scheme. For clarity, non-polar H atoms, water molecules, and solvated cations are hidden, while non-guanine atoms of HA-functionalized G-quadruplex are shown as lines in (b)-(i). (j) Comparison of the relative weight of guanine···guanine hydrogen bonds (N1–H···O=C in red, N–H_2_···N7 in blue, and N1–H···N7 in magenta) during the concatenated last 10 ns of the MD simulations of all systems.

Each G4, built with four guanine bases surrounding a cation (illustrated with K^+^ in Figure 2a), was repeated four times, affording a HA-functionalized G-quadruplex model (Figures 2b and S2, SI). The dynamic behavior of each G-quadruplex–cation complex was then evaluated through three independent MD runs of 50 ns. Illustrative snapshots of the MD simulations with K^+^, Li^+^, NH_4_^+^, Ca^2+^, Na^+^, Mg^2+^, and Cs^+^ are presented in Figure 2c–i. The outermost cation in the G-quadruplex systems assembled by K^+^, Na^+^, NH_4_^+^, Mg^2+^, and Ca^2+^ was quickly solvated, with concomitant loss of the M···O bonding interactions, leading to a ratio of four G4 to three intercalated cations. In contrast, the four smaller Li^+^ cations remained coordinated by their G4’s four carbonyls oxygen atoms until the end of the MD simulations (Figure 2d). On the other hand, the larger Cs^+^ ions disrupted the G-quadruplex, leading to a partial G4 disassembly, with two cations hosted between G4 and the other two being hydrated. Moreover, in the MD simulations with the Ca^2+^ binder, the cation’s charge led to repulsions that pushed two opposite Ca^2+^ to the top and bottom of the G-quadruplex, without disrupting it (Figure 2f). In the MD simulations with Mg^2+^, with a radius close to Li^+^,^32,56^ but with higher charge density, the divalent cation induced the loss of the coplanarity between the guanine bases in each G4 (Figure 2h). The impact of each coordinated cation on the stability of the G-quadruplexes is illustrated in the SI, Movies S1–S7, in ref (62) for selected MD runs.

Typical geometric descriptors for DNA-based G-quadruplexes, such as Root-Mean-Square-Deviations and Radius of Gyration of the G4, were assessed. However, as reported in the SI (Section 3.1, SI), they were rather insufficient to characterize and differentiate between the supramolecular assemblies. Thus, there was the need to use other descriptors to evaluate the impact of the different cations on the stability of these G-quadruplex systems. Each G4 in a DNA-based G-quadruplex assembles *via* up to 8 ideal cooperative Hoogsteen-type hydrogen bonds: four internal N1–H···O=C interactions, and four more external N–H_2_···N7 interactions (Scheme S1, SI).^61,63^ Therefore, in each simulated G-quadruplex system, a total of up to 16 N1–H···O=C and 16 N–H_2_···N7 putative interactions are expected (see Scheme S1, SI for the standard atomic numbering of G). The evolution of the number of hydrogen bonds (see Section 3.1 in the SI) between adjacent guanine moieties (regardless of the G4 they belong to) along the various MD runs is plotted in Figure S5 (SI), while the statistics for the concatenated last 10 ns of each MD run for each cation are gathered in Table S3 (SI). The structural features found in the MD simulations described above are translated by the differences in the pattern of hydrogen bonding interactions, with a significant impact on the N1–H···O=C hydrogen bonds between guanine bases. These bonds are almost absent with Ca^2+^ and Mg^2+^, very low with Li^+^ (*ca.* 3), and moderate with Na^+^ (*ca.* 5) and Cs^+^ (*ca.* 6). Only with K^+^ and NH_4_^+^ at least 9 N1–H···O=C hydrogen bonds are maintained, but still distant from the ideal of 16. In contrast, the N–H_2_···N7 hydrogen bonds are less affected, with average values close to 9, except for Ca^2+^ and Cs^+^ (*ca.* 5) and Mg^2+^ (*ca.* 1). A third type of relevant hydrogen bonding interactions within G4, N1–H···N7 (Scheme S1, SI), is also observed, with average values up to 8 in the MD simulations of Mg^2+^ (Table S3, SI). The relative weight of the three types of interactions is plotted in Figure 2j, showing that the increase of N1–H···N7 interactions is accompanied by the decrease of the N1–H···O=C ones, being particularly evident for the divalent Mg^2+^ and Ca^2+^ cations. We hypothesize that these results could be a consequence of the reorientation of the guanine bases’ oxygen atoms to accomplish the geometric coordination requirements of each cation binder.

The interactions between the different cation binders and the carbonyl groups in the guanine bases were evaluated through M···O distances, M···O=C angles, and the total number of M···O interactions (see Section 3.1 in the SI), observed for the last 10 ns of the three independent runs carried out for each G-quadruplex system (30 ns of sampling data). Their values are listed in Table S4 in the SI, together with the number of intercalated cations (*vide supra*) and their corresponding coordination numbers (CN), calculated as the ratio between the total number of M···O interactions and the number of intercalated cations. As expected, the M···O distances reflect the sizes of the cations. In the MD simulations with K^+^, NH_4_^+^, and Na^+^ intercalated between G4 and surrounded by eight carbonyl groups, K^+^ and NH_4_^+^ have CN of *ca.* 7, whereas the CN of Na^+^ is only 5.4, with the Na^+^···O interactions being more intermittent. However, the average number of NH_4_^+^···O=C hydrogen bond analyses is 4.3 (average of 12.85 ± 1.36 for the three cations), being necessarily lower than its CN due to the additional angle cut-off criterion. On the other hand, the high CN of 6.5 for larger Cs^+^ is misguiding, considering that the two intercalated cations were only able to sustain three G4. The CN of 4 for Li^+^ is self-explanatory, as each cation is surrounded by only the four oxygen atoms of a single G4. Despite their different sizes and the different representative binding scenarios, both Mg^2+^ and Ca^2+^ afford CNs of 5.3. The M···O=C angles showed that the guanine bases align more linearly with Mg^2+^ and Ca^2+^ than with the lower density charged Li^+^ and Na^+^ ions. Most likely, this is due to the great distortion of G4s from planarity when coordinating the divalent cations (*vide supra*).

The distances between centers of mass of neighboring G4 (Table S5, SI) were assessed as surrogates for the *π–π* stacking interactions. Apart from Cs^+^, the average values of G4···G4 distances, ranging between 3.54 ± 0.12 (Na^+^) and 4.11 ± 0.20 Å (Mg^2+^), are consistent with the existence of*π–π* stacking interactions, despite the small distortions induced by the different cations. Indeed, these values are close to the G4···G4 distances of 3.6 Å observed in DNA-based G-quadruplexes.^64^ The G4···G4 average distances in the systems with Cs^+^ are 6.18 ± 3.25 Å, reflecting the instability caused by the absence of a third binder assembling the fourth G4. On the other hand, the distances between G4 with intercalated Cs^+^ cations are only 3.86 ± 0.56 Å, falling within the range given above for the remaining binders and marginally reflecting the larger size of this alkali metal cation.

Overall, the structural insights obtained with the preliminary MD simulations carried out with the HA-functionalized G-quadruplex model showcase that K^+^ and NH_4_^+^ have a better fit to the assembled G4 and, thus, a higher ability to mediate the assembly of the HA-functionalized G-quadruplex supramolecular hydrogel, corroborating the experimental findings. Only these cations were shown to properly intercalate between G4, while being shared by nearly all the eight available carbonyl oxygen atoms. Moreover, they have the least impact on the ancillary hydrogen bonding framework, essential for stabilizing each G4. Therefore, the distorted binding arrangements observed in the MD simulations with Li^+^, Na^+^, Cs^+^, Mg^2+^, and Ca^2+^ indicate that the gelation ability of the HA-functionalized G-quadruplex supramolecular hydrogel should be reduced when compared with the K^+^ or NH_4_^+^ ions.

### Physicochemical and Morphological Characterization of the G-Quadruplex Self-Assembled Hydrogels

**Figure 3:**
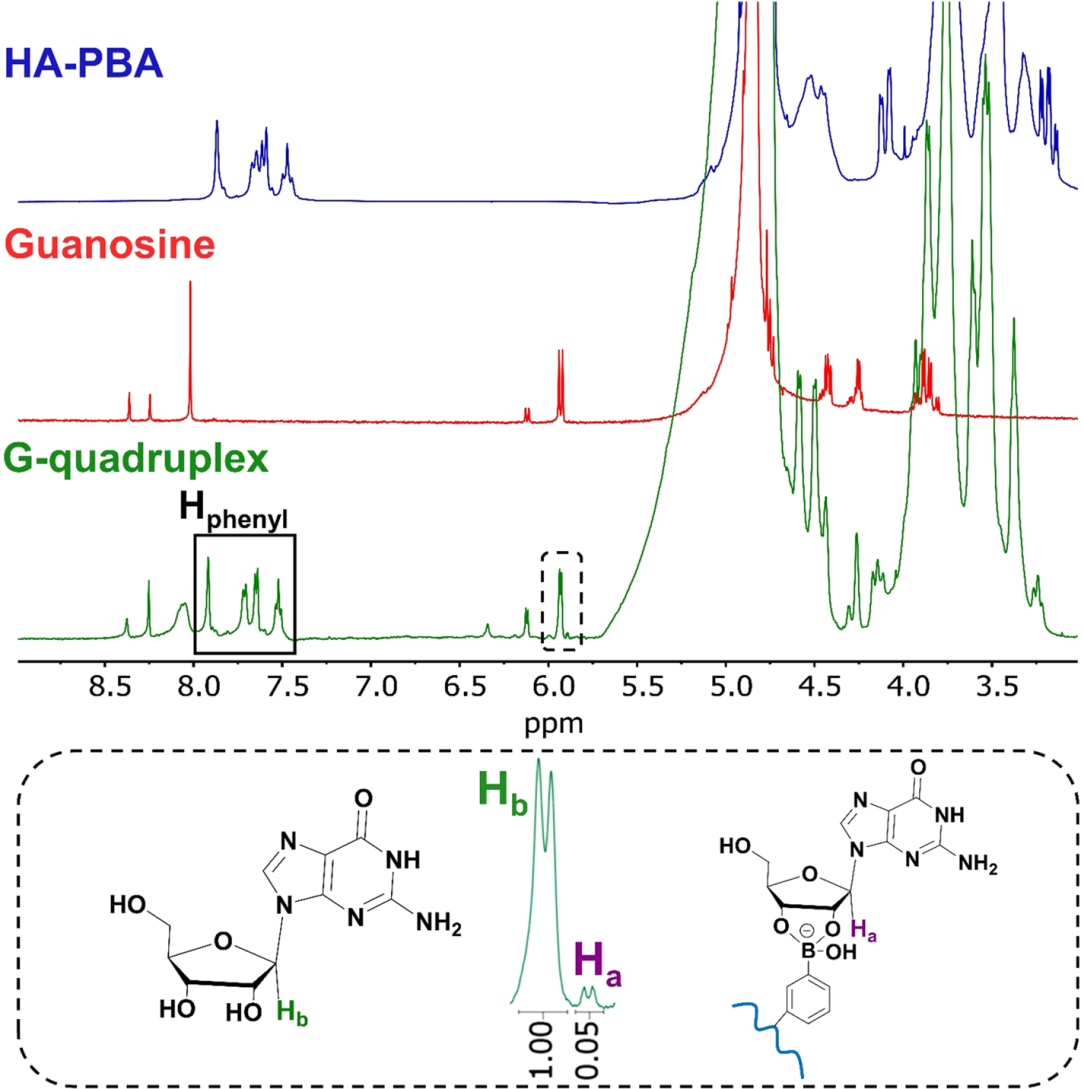
^1^H NMR spectra of HA-PBA (blue line), guanosine (red line), and 1.5% (w/v) HA-functionalized G-quadruplex hydrogel (green line) at 25 °C in D_2_O at pH = 7.4.

The formation of dynamic boronate ester bonds (B–O–C), fundamental in the formation of K^+^-templated dynamic G-quadruplex supramolecular hydrogels, was unveiled by ^1^H NMR spectroscopy (Figure 3) through the presence of a doublet signal at δ = 5.89 ppm (*J* = 3.9 Hz), corresponding to the H_a_ proton.^21,33^ Moreover, a doublet signal was also denoted at δ = 5.93 ppm (*J* = 5.7 Hz), assigned to the proton of unreacted G (H_b_). The integration of both H_a_ and H_b_ proton peaks provides information about the ratio between reacted and unreacted G. Although, in this study, only 5% of G reacted, such behavior was expected since the formation of these dynamic bonds is favored at basic conditions (above the p*K*_a_ of the PBA), but difficult to accomplish at physiological pH.^65−67^

**Figure 4:**
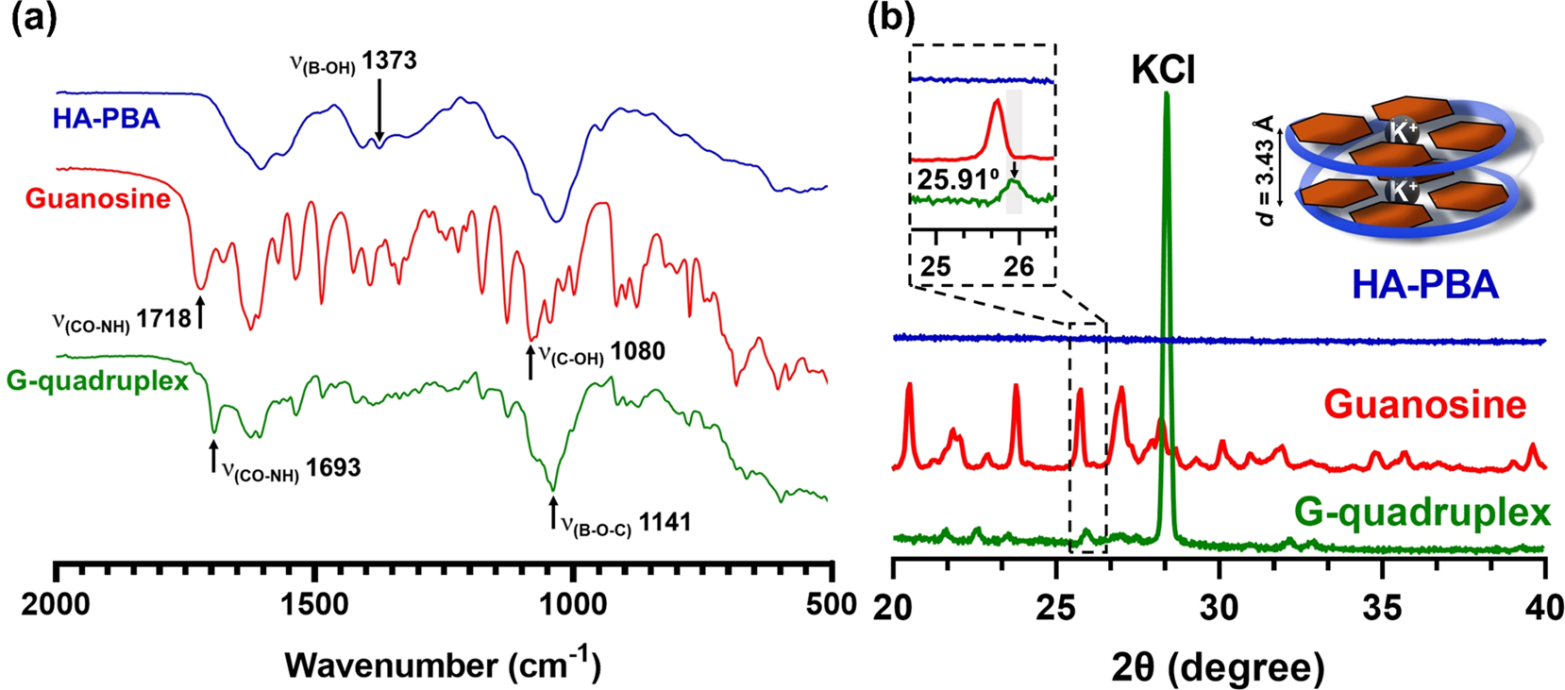
(a) ATR-FTIR and (b) PXRD spectra of HA-PBA (blue line), guanosine (red line), and HA-functionalized G-quadruplex freeze-dried hydrogel (green line).

To better elucidate the key role of the solution pH in the formation of the boronate ester bonds, we performed a ^1^H NMR study under acidic (pH = 4) and basic (pH = 10) conditions (Figure S6, SI). In this regard, it is evident that acidic environments hinder the formation of boronate ester bonds, since the amount of reacted G species (H_a_) is vestigial, thus do not enabling the formation of a gel. On the other hand, at basic pH, the absence of the unreacted G proton peak (H_b_) is denoted, given that almost all G established boronate ester bonds, thus confirming that alkaline environments favor the formation and stability of intramolecular boronate ester bonds.^68^ The formation of the boronate ester bonds was also confirmed by ATR-FTIR spectroscopy (Figure 4a). The absence of the typical vibration band of free boronic acid diols (ν_B–OH_) at 1373 cm^–1^ in the ATR-FTIR spectrum of the G-quadruplex hydrogel suggests the transformation of the free boronic acid diol groups into boronate ester bonds *via* the reaction of the boronic acid groups on HA-PBA with the *cis*-1,2-diols of G. This was indeed confirmed by the appearance of a new vibration band at 1141 cm^–1^ (ν_B–O–C_).^18^ Furthermore, the fact that the vibration band of the G-free diols (ν_C–OH_ = 1080 cm^–1^) was not present in the G-quadruplex hydrogel indicates the formation of cyclic boronate ester bonds within the hydrogel network. In addition, due to the intramolecular hydrogen bonding within the G4 structures, the amide vibration band of G shifted from 1718 to 1693 cm^–1^ in the hydrogel.^30^

PXRD was also used to validate the formation and microstructure of the G-quadruplex supramolecular structures in the hydrogel by unveiling the presence of *π–π* stacking interactions between the G4, as well as the distance (*d*) between them.^31,45,69,70^ The PXRD pattern of the freeze-dried HA-functionalized G-quadruplex hydrogel showed a characteristic Bragg diffraction peak at 2θ = 25.91°, corresponding to a *d* = 3.43 Å, which is consistent with the*π–π* stacking interactions between two adjacent planar G4 entities held together by K^+^ ions (Figure 4b). The PXRD patterns of HA-PBA and G were also recorded and did not show any peak at 2θ = 25.91°, thus confirming the formation of the G-quadruplex supramolecular structures in the hydrogel. In fact, the G4···G4 distance fits the range of [3.21:4.20] Å calculated for the MD simulations with K^+^ (Table S5, SI).

**Figure 5:**
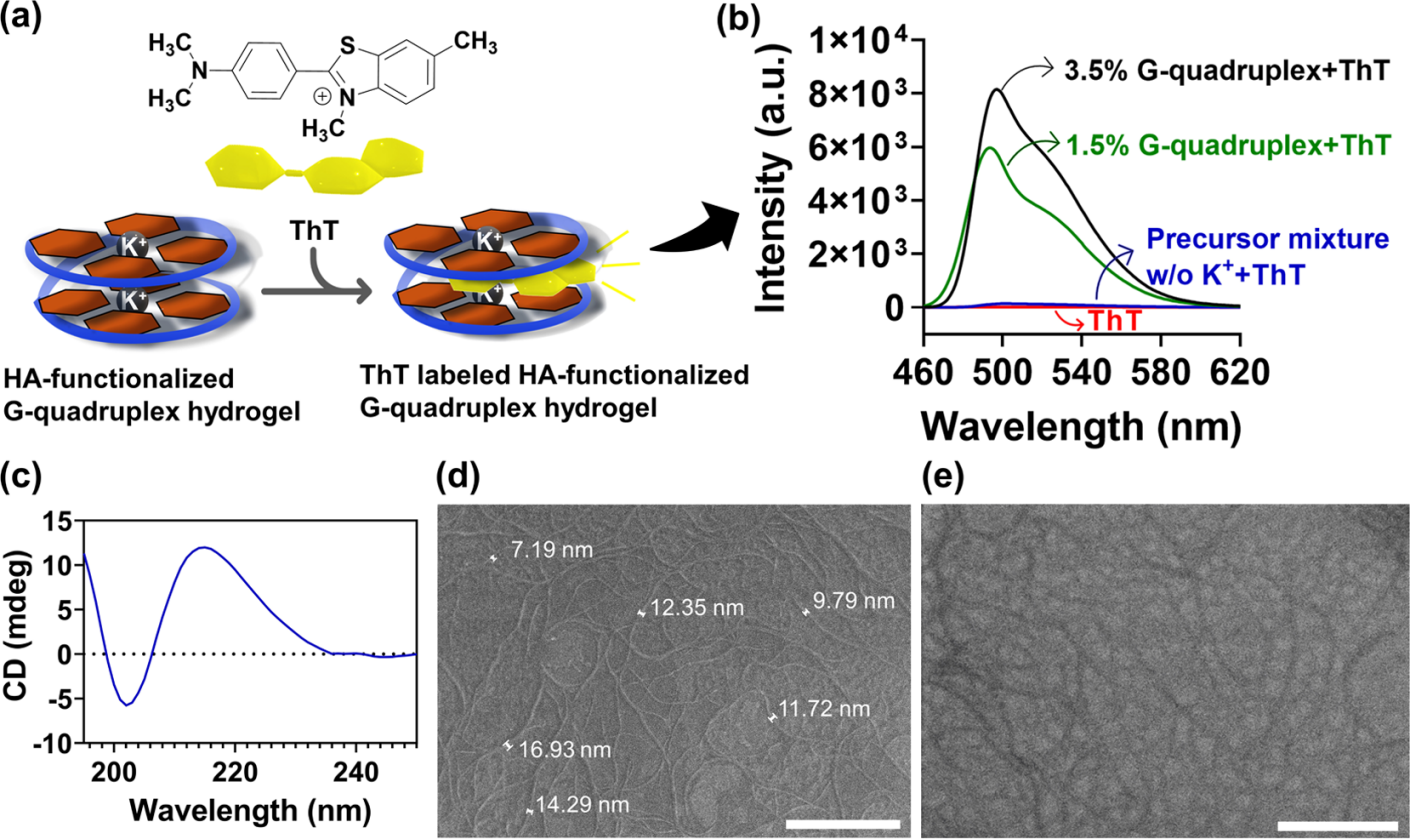
(a) Schematic representation of the mechanism for the formation of ThT-labeled fluorescent HA-functionalized G-quadruplex hydrogel. (b) Fluorescence spectra of bare ThT solution (red line), ThT-incorporated precursor mixture without K^+^ (blue line), and ThT-labeled HA-functionalized G-quadruplex hydrogel at 1.5 (green line) and 3.5% (w/v) (black line). (c) CD spectrum of HA-functionalized G-quadruplex hydrogel. (d) SEM and (e) TEM micrographs of the HA-functionalized G-quadruplex hydrogel. Scale bars: 500 nm.

The highly intense and sharp diffraction peak at 2θ = 28.38° (*d* = 3.14 Å) is assigned to the crystalline KCl, as described elsewhere.^71^ The formation of the G-quadruplex structures was further confirmed by fluorescence spectroscopy using ThT as a fluorescence probe (Figure 5a). ThT is a hydrophilic cationic dye which has a strong and selective affinity to G-quadruplex supramolecular structures, enhancing its fluorescence intensity, thus being widely used to monitor the formation of G-quadruplex structures in hydrogels.^26,72^ Indeed, once bound to the K^+^-templated G-quadruplex self-assembled structures either at 1.5 or 3.5% (w/v), the fluorescence intensity of the ThT-loaded G-quadruplex hydrogel experienced a red-shift and increased significantly by 500-fold (peak at 493 nm) and 700-fold (peak at 497 nm), respectively, when compared with the very weak fluorescence emission peak of the ThT solution (1 mM) alone centered at 490 nm. Such behavior reveals the *π–π* stacking interactions between the K^+^-templated G4 structures and ThT, and the formation of the G-quadruplex self-assembled structures (Figure 5b).^26,72,73^

Moreover, the higher intensity peak, *i.e.,* enhanced fluorescence intensity obtained for the 3.5% (w/v) hydrogel when compared to the 1.5% (w/v) hydrogel reveals a higher cross-linking density, binding, and extent of *π–π* stacking interactions between ThT and G4 monomers in the former hydrogel. On the other hand, although adding ThT to a precursor mixture lacking K^+^ slightly improved the fluorescence emission peak of ThT, it is negligible when compared to the fluorescence emission peak of ThT after binding to the K^+^-templated G-quadruplex hydrogels. These findings prove the importance of K^+^ in the formation and stabilization of the G-quadruplex self-assembled structures. Moreover, CD spectroscopy was used to assess the secondary structure and conformation of the G-quadruplex self-assembled structures. CD bands between 200 and 300 nm have been reported as a critical region to assess the secondary structure of G-quadruplexes.^74^ Typically, a positive band at ∼265 nm and a negative band at ∼240 nm are representative of a parallel G4 arrangement, while a positive band at ∼290 nm and a negative band at ∼260 nm are associated with an antiparallel structure.^4,9^ However, differences in the G4 stacking, segment orientation, and loop arrangement dictate changes in the position of the peaks and, thus, on the G-quadruplex secondary structure.^75^ Moreover, the concentration of the K^+^ ion can also induce conformational changes in the G-quadruplex secondary structure.^76^ As shown in Figure 5c, the HA-functionalized G-quadruplex supramolecular hydrogel exhibited a negative peak at 202 nm and a positive peak at 215 nm, which is assigned to the *n*–π* transition of the G amide moiety.^18,77,78^ The overall CD spectrum suggests that, although there is a blue-shift in the characteristic bands of the G-quadruplex self-assembled structures, it mostly adopts a parallel secondary structure.

The morphology of the HA-functionalized G-quadruplex hydrogel was investigated by STEM. Both the SEM (Figure 5d) and the TEM micrographs (Figure 5e) showcased the existence of a uniform and entangled 3D nanofibrillar network in the hydrogel with several micrometers in length and a few nanometers in width (12 ± 5 nm). The aggregated nanofibrous network suggests the stacking of several G4 monomers, which self-assemble to form the G-quadruplex self-assembled fibrillar structure.^25,44,79^ The physical cross-linking, branching, and aggregation of the nanofibers drove the gelation process by entrapping large amounts of water molecules, thus enabling the formation of G-quadruplex supramolecular hydrogels whose robustness was assessed by measuring their rheological properties.^16,18^

### Rheological Properties of the G-Quadruplex Hydrogels

**Figure 6:**
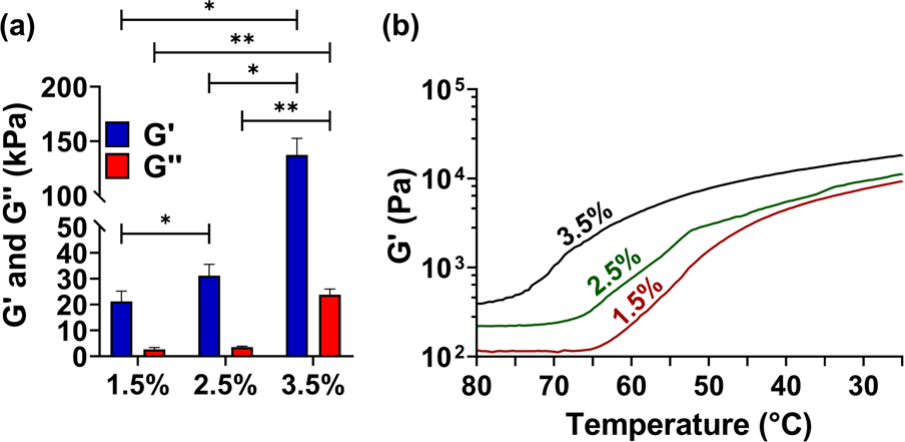
Rheological properties of HA-functionalized G-quadruplex supramolecular hydrogels. (a) *G*′ and *G*″ values at 1 Hz and 25 °C as a function of the hydrogel concentration, *p < 0.05 (relative to *G*′), **p < 0.05 (relative to *G*″). (b) Evaluation of the storage moduli of the hydrogels as a function of the temperature.

The viscoelastic properties of the G-quadruplex hydrogels at 1.5, 2.5, and 3.5% (w/v) were investigated by rheology. Oscillatory amplitude sweep experiments were performed at RT to determine the linear viscoelastic region (LVR), where the storage modulus *G*′ (*i.e.,* elastic modulus) and the loss modulus *G*″ (*i.e.,* viscous modulus) are independent of the shear strain. As shown in Figure S7a, SI, all hydrogel formulations exhibited a LVR from 0.1 to approximately 1% strain, and a value within this region was chosen to conduct further tests. At all tested hydrogel concentrations and throughout the experiment, the hydrogels exhibited a crossover point, although at different strain values, in which the loss modulus *G*″ surpassed the storage modulus *G*′, which reveals the disruption of the cross-linked polymeric network. The frequency behavior of both dynamic moduli was also evaluated in the range of 0.1 to 10 Hz at 25 °C. The constructs displayed a minor frequency-dependent viscoelastic behavior within this frequency range, with the *G*′ being higher than the *G*″ for all studied concentrations, thus revealing the gel-like character (Figure S7b, SI). The influence of the hydrogel concentrations on the mechanical strength of the fully formed hydrogels was also evaluated by comparing the mean *G*′ and *G*″ moduli values at a constant frequency strain of 1 Hz and at a constant temperature of 25 °C (Figure 6a). The increase in the hydrogel concentration from 1.5 to 3.5% (w/v) led to an increase of the *G*′ values from 21 to 137 kPa, thus indicating the formation of stronger hydrogels. A similar trend was observed for the *G*″. As expected, the stiffness of the HA-functionalized G-quadruplex hydrogels improved while increasing the concentration of the polymer, owing to the increment of dynamic covalent bonds formation, thus, a higher cross-linking density, as well as a higher amount of *π–π* stacking interactions between G4 monomers, as corroborated by the ThT assay. To ascertain the influence of the temperature on the hydrogelation mechanism, temperature sweep tests were attempted (Figure 6b). The decrease of the temperature to 25 °C triggered a sol–gel transition, as evidenced by the increase in the *G*′ from *ca.* 500 to 9000 Pa. The *G*′ values in the temperature sweep tests (Figure 6b) are *ca.* 2 orders of magnitude lower than the ones obtained in the frequency sweep assays (Figure 6a) since, in the former, the hydrogelation process occurred *in situ* while varying the temperature, giving information about the gelation kinetics.

### Shear-Thinning and Self-Healing Properties

**Figure 7:**
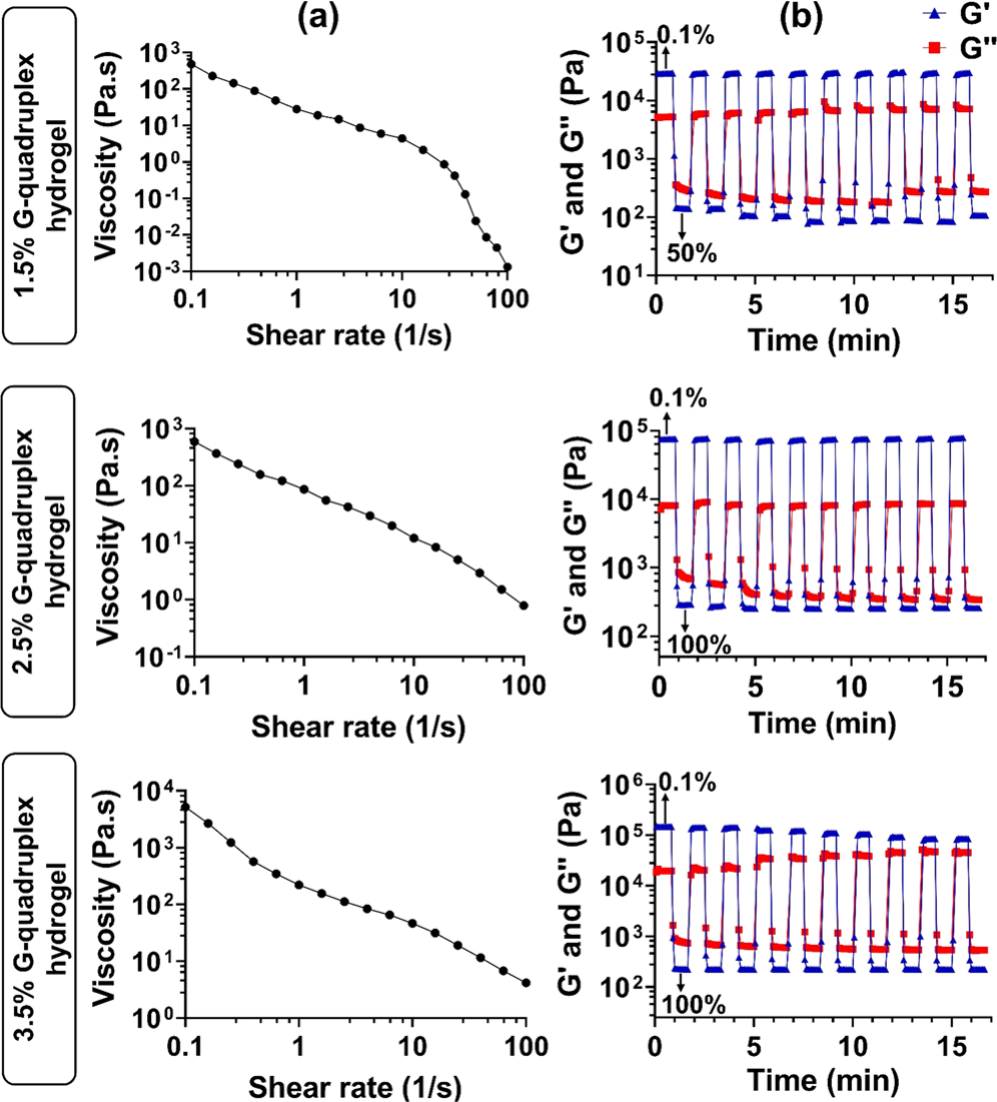
(a) Viscosity and shear-thinning behavior of HA-functionalized G-quadruplex supramolecular hydrogels. (b) Dynamic oscillatory tests at 1 Hz by employing alternate strain values from 0.1 to 50/100%.

Supramolecular self-assembly and dynamic covalent cross-linking are inherently linked to shear-thinning and self-healing properties, which are common features of dynamic G-quadruplex supramolecular hydrogels.^18,25,29,43,80,81^ In this regard, the viscosity profile of the hydrogels at increasing shear rates was studied. As shown in Figure 7a, the viscosity of the hydrogels, at all concentrations studied, decreased as the shear rate increased, which is a characteristic non-Newtonian shear-thinning behavior, a key aspect for injectability. In addition, oscillatory tests were carried out to evaluate the autonomous recovery (*i.e.,* self-healing behavior) of the HA-functionalized G-quadruplex hydrogels to their original mechanical properties after applying several step strain cycles (Figure 7b).

Thus, alternative steps of 0.1% (low) and 50/100% (high) strains were applied on the different hydrogel formulations over a period of around 17 min and ten cycles. The high strain values for each hydrogel concentration were chosen according to the amplitude sweep experiments as the highest possible value (larger deformation) after the crossover point in which the disruption of the 3D cross-linked polymeric network was observed. Upon application of a low strain value (0.1%) within the LVR, all hydrogels maintained their gel-like character (*G*′ > *G*″), whereas at a high strain (50/100%), where *G*″ modulus is superior to *G*′, the hydrogels experienced a transient gel–sol phase transition (*i.e.,* polymer network disruption), thus losing their viscoelastic properties. However, when the low strain was reapplied, the hydrogels displayed a fast recovery (∼1 min) to their original *G*′ values (gel state), which became higher than the *G*″ values, due to the re-establishment of the dynamic cross-links, thus recovering their original microstructure and mechanical properties. Briefly, the HA-functionalized G-quadruplex hydrogels at 1.5 and 2.5% (w/v) fully recovered (*ca.* 100%) their original *G*′ values after one deformation cycle, which remained unchanged until the last cycle, thus revealing their remarkable self-healing behavior. On the other hand, the 3.5% (w/v) hydrogel showed a high recovery rate after the first disruption cycle (*ca.* 95%), which decreased up to *ca.* 60% after repeating ten strain cycles, thus revealing the partial recovery of the hydrogel’s original microstructure. These results highlight the outstanding self-healing properties of the G-quadruplex hydrogels, mainly those at 1.5 and 2.5% (w/v). The water content of the hydrogels can significantly improve their biocompatibility, as well as mimic the highly hydrated microenvironment of the native ECM of biological tissues.^82−84^ Having this in mind, the water content (%) of the HA-functionalized G-quadruplex hydrogels was measured by weighing the hydrogels right after their preparation and after freeze-drying (Figure S8, SI). For all tested concentrations, the water content values ranged between 88 and 94%. Notably, the percentage of water decreases as the concentration increases. It has been reported that hydrogels with higher stiffness generally have lower water content.^84,85^ Our results agree with previous reports since the 3.5% (w/v) hydrogels, which contain the low water content (*ca.* 88%), denoted a higher elastic modulus and hence, enhanced gel strength. Additionally, the presence of water molecules confined in the hydrogels can readily participate in self-hydrogen bonding and, thus, enhance and accelerate the self-healing process.^86,87^ In this regard, the lowest self-healing ability was attained by the 3.5% (w/v) hydrogel (Figure 7b).

**Figure 8:**
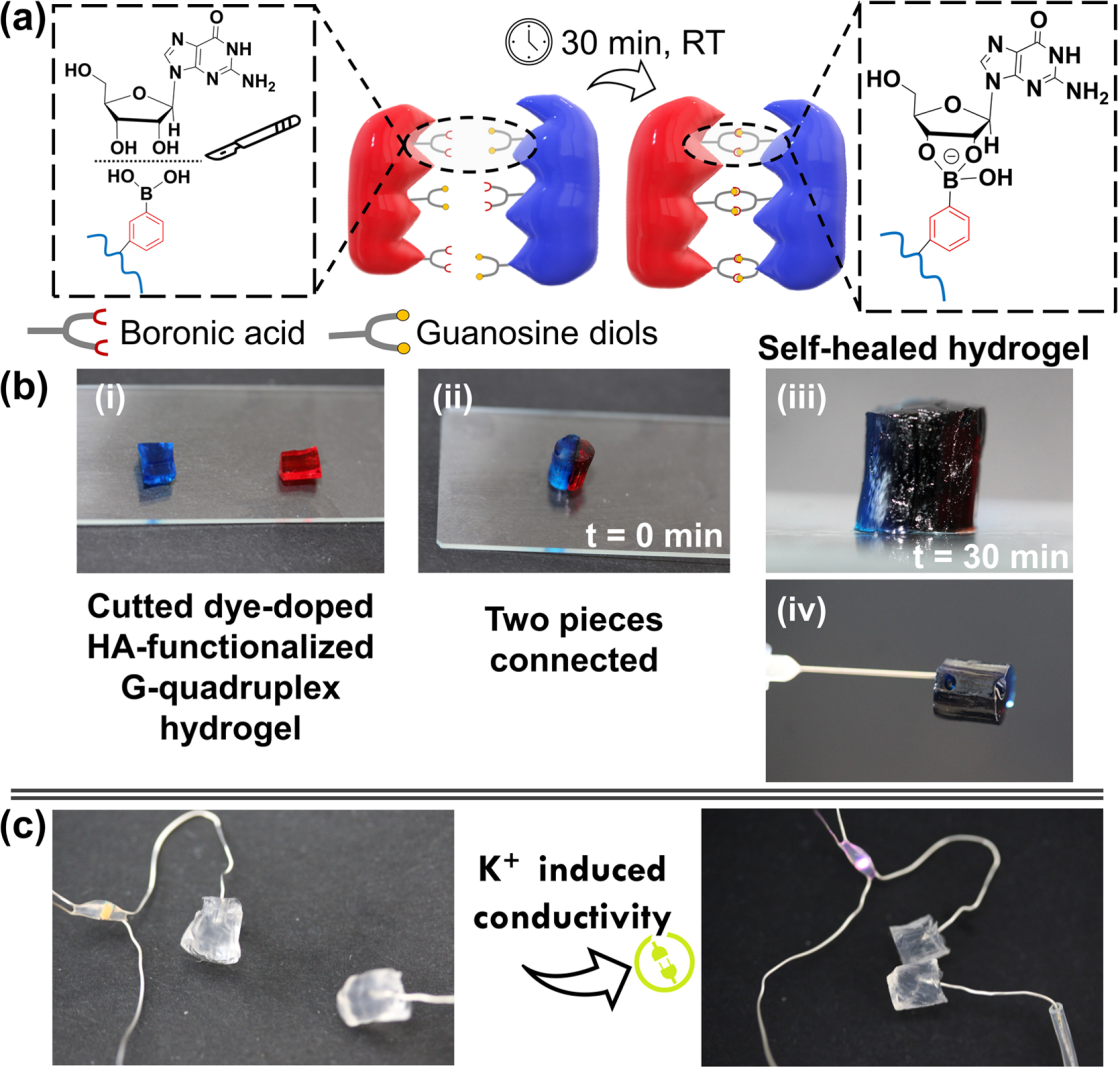
(a) Schematic illustration of the self-healing process due to the dynamic nature of the *in situ* cyclic dynamic boronate ester bonds established between the guanosines and phenylboronic acid moieties. (b) Digital images revealing the self-healing behavior of the HA-functionalized G-quadruplex hydrogel after being cut into two pieces, stained with different dyes (i), and promoting their connection for 30 min at RT (ii, iii); the self-healing behavior was also assessed by a gravity-defying test using a needle (iv). (c) Digital images demonstrating the conductivity of the HA-functionalized G-quadruplex using an electrode-based LED system.

The self-healing behavior of the dynamic G-quadruplex supramolecular hydrogels, formed by covalent reversible cyclic boronate ester bonds was also assessed following the procedure depicted in Figure 8a. Figure 8b and SI, Movie S8, in ref (62) clearly show that, after cutting the hydrogel in two pieces, each one stained with a different dye (Figure 8b-i) and promoting their contact for 30 min at RT (Figure 8b-ii), the hydrogel was completely healed into a single, integral piece without the need for any external stimuli (Figure 8b-iii,iv). Furthermore, it was evident the diffusion of both dyes toward the rejoined region of the healed hydrogel (Figure 8b-iii), which maintained its integrity even after heavy shaking in water at 100 rpm, clearly showing that the two pieces were well-bonded (see SI, Movie S8, in ref (62)). This visual insight was supported by the rheological data, thus revealing the remarkable self-healing behavior of the HA-functionalized G-quadruplex hydrogels.^37^

### Injectable Properties

We have assessed the injectability of all hydrogel formulations, which is an important and common feature of dynamic supramolecular hydrogels that enable their extrusion with minimal external effort into target sites. In fact, dynamic and injectable supramolecular hydrogels that could be introduced in the human body*via* minimally invasive procedures and adapt on-demand to target-specific sites are highly desired in bioapplications, including in drug/therapeutics delivery, tissue engineering and regenerative medicine. As such, the injectability of the G-quadruplex hydrogels was assessed with different needle gauges (16, 21, and 25 G; Figure S9, SI). We hypothesize that, besides hydrogen bonding and *π–π* stacking interactions between the G-quartets, dynamic boronate ester bonds also contribute to their injectability.^27,73^ In fact, all hydrogel formulations presented good injectability, with excellent volume stability and minimal water leakage, although being dependent on the needle gauge. As disclosed in Figure S9 (SI), the 1.5% (w/v) hydrogel was injectable through all used needle gauges since it presents a lower stiffness. However, the same did not occur for the stiffer hydrogels at 2.5 and 3.5% (w/v), whose injectability could not be accomplished with the 25 G needle. As expected, the mechanical strength of the hydrogels strongly influences their injectability. Moreover, due to their mechanical strength, the hydrogels acquired a well-defined needle-shaped structure that can be easily handled (see SI, Movie S9, in ref (62)).

### Thermoresponsive and Conductive Properties

Cross-linked hydrogen bonded supramolecular structures are known to assign thermoresponsive properties to biomaterials due to their temperature-dependent reversible assembly/disassembly.^88^ In this work, we have assessed the thermoresponsiveness of the HA-functionalized G-quadruplex hydrogels by alternatively applying repetitive cooling/heating cycles (see SI, Movie S10, in ref (62)). Upon cooling to RT, gelation takes place, corroborating the temperature sweep rheological test. However, by heating the hydrogel to 80 °C, it was possible to observe the complete fusion of the gel, which experienced a gel–sol phase transition due to the disassembly of the K^+^-templated G4 structures caused by the disruption of the hydrogen bonds at higher temperatures.^89^ This process was repeated at least twice without affecting the hydrogel formation capability. Besides, G-quadruplex hydrogels denoting a high content of metal cations have proven to be conductive.^30,88^ Having this in mind, and making use of a battery-powered circuit wired to a LED indicator, it was possible to demonstrate the conductivity of the G-quadruplex supramolecular hydrogel upon putting the two pieces together, clearly denoted by the LED turning on (Figure 8c, see SI, Movie S11, in ref (62)), holding great potential to be used in bioelectronics.

### Fabrication of Perfusable Microchannels within Photo-Cross-Linkable Supporting Matrices

**Figure 9:**
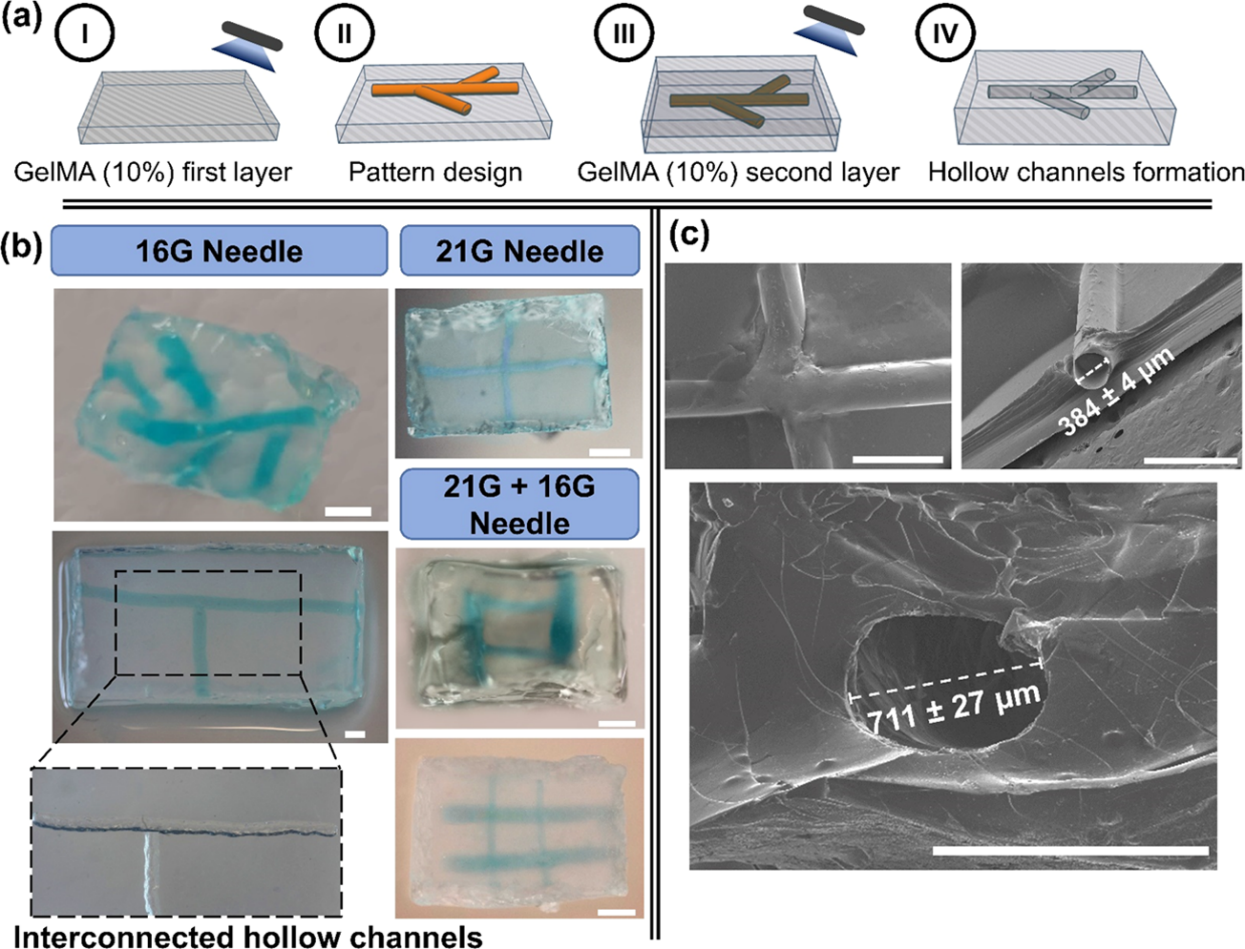
(a) Schematic representation of the employed strategy to engineer the GelMA 10% (w/v) constructs embedding the perfusable hydrogels: I. 10 s pre-photopolymerization step to increase the robustness of the GelMA first layer; II. Fiber’s arrangement in the desired pattern; III. GelMA second layer pouring and full photopolymerization; IV. Fiber’s degradation overnight at 37 °C in culture medium/PBS. (b) Optical photographs of the well-defined and tunable-size hollow microchannels filled with blue dye. Scale bars: 2 mm. (c) SEM micrographs of the interconnected hollow microchannels within the GelMA construct. Scale bars: 1 mm.

As aforementioned, G-quadruplex hydrogels are rather unstable.^45,90^ In fact, different sources of potassium cations can influence the G-quadruplex’s stability. For instance, it has been reported that KCl-based G-quadruplex hydrogels are less stable than KOH counterparts, resulting in the precipitation of G and hydrogel decomposition within a few hours.^25^ However, while using KOH, the pH behind the production of the hydrogel (8–9) is too high to ensure its cytocompatibility, which is a major bottleneck when aiming for bioapplications.^32^ Hence, we hypothesized that the HA-functionalized G-quadruplex hydrogels, assembled at physiological pH, could allow the production of perfusable microchannels, embedded in larger bulk hydrogels, for enabling the diffusion of nutrients and oxygen, essential for sustaining cell survival.^49,91^ Despite the overnight self-degradation of the G-quadruplex hydrogel when immersed in aqueous solutions (PBS/culture medium) at 37 °C (see SI, Movie S12, in ref (62)), the hydrogel has a lifetime stability of 2 weeks at RT. As such, the well-defined needle-shaped fibers obtained after the hydrogel’s extrusion were embedded in a biocompatible supporting GelMA matrix at 10% (w/v), on a PDMS mold, aiming to develop hollow microchannels (Figure 9a). Due to the hydrogel fiber’s stiffness, they could be easily handled and placed in the desired pattern (Figure 9b). After photopolymerization of the GelMA pre-hydrogel aqueous solution, embedding the G-quadruplex supramolecular hydrogel, and further incubation of the full 3D construct overnight in culture medium at 37 °C, well-defined, size- and shape-tunable hollow microchannels were obtained within the 3D GelMA supporting matrix.

To overcome the influence of the viscosity of the polymer matrix, which could be an issue regarding the placement of the fibers, a 10 s pre-photo-cross-linking step was attempted before the fiber’s arrangement. This pre-photopolymerization step was essential to increase the robustness of the supporting matrix first layer. To ensure that this preliminary step only corresponds to a small part of the total photo-cross-linking process, a gel point rheology test was performed with GelMA 10% (w/v) at 25 °C and the shear strain was fixed within the LVR (Figure S10a, SI). Figure S10b (SI) allows us to conclude that the pre-photo-cross-linking step (10 s) resulted in an increase of *G*′ of around 10-fold, whereas the final photo-cross-linking (290 s) promoted the highest increase of *G*′ (around 50-fold). These results suggest that we were able to increase the robustness of the GelMA’s first layer to sustain the fibers without compromising the photo-cross-linking of the final 3D construct. Moreover, we were able to create perfusable microchannels embedded in different methacrylated natural polymer supporting matrices, with no associated viscosity, demonstrating the versatility of the proposed methodology (Figure S11, SI). Furthermore, such an approach enables the production of microchannels, with tunable size and geometry, in a straightforward manner by the simple incubation of the 3D construct in PBS or culture medium at 37 °C.

The SEM images (Figure 9c) clearly revealed the formation of well-defined and interconnected hollow microchannels denoting inner diameters of either 384 ± 4 μm or 711 ± 27 μm while resorting to needles with 21 G or 16 G, respectively. Additionally, the perfusion extension and interconnectivity of the hollow microchannels were confirmed by the successful flow of a blue dye aqueous solution injected through the microchannels (Figure 9b, see SI, Movie S13, in ref (62)).

### *In Vitro* Cell Viability

**Figure 10:**
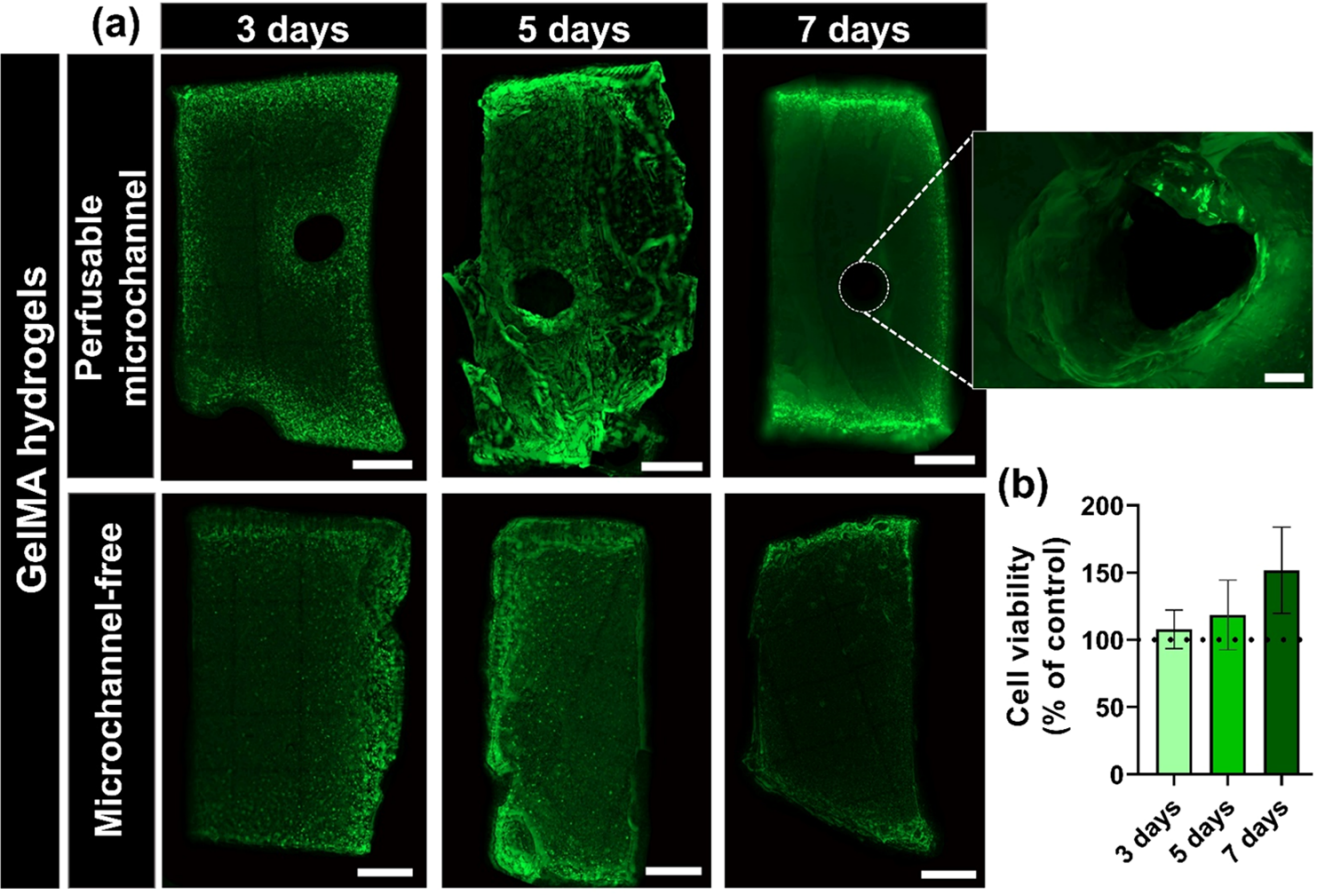
(a) Live/dead fluorescence microscopy cross-section micrographs of hASCs encapsulated in a microchannel-free (control) and perfusable microchannel embedded in a 10% (w/v) GelMA hydrogel (sample) at 3, 5, and 7 days of culture. Live cells are stained with calcein-AM in green and dead cells are stained with PI in red. Scale bars: 1000 μm, inset scale bar 200 μm. (b) Cell viability quantification, expressed as a percentage of the control, of hASCs encapsulated in the perfusable microchannel embedded in the GelMA hydrogel for 3, 5, and 7 days of culture.

To validate the effectiveness of the perfusable microchannels to be used as platforms to enable the diffusion of nutrients and oxygen in larger hydrogels to sustain long-term cell viability, an *in vitro* live/dead biological assay was conducted. For this purpose, a HA-functionalized G-quadruplex fiber prepared with a needle of 16 G was embedded in a human adipose stem cells (hASCs) laden GelMA supporting matrix (L × W × H; 10 × 8 × 5 mm) at a density of 2 × 10^6^ cells/mL. A microchannel-free bulk 3D construct was used as a control. Both 3D constructs were prepared with larger dimensions to better mimic the human tissues in which the cells search for blood vessels to obtain nutrients and oxygen. The constructs were incubated in cell culture medium for 3, 5, and 7 days and the cell viability monitored by fluorescence microscopy (Figure 10a).

The live/dead staining of a cross-section of the 3D construct embedding the perfusable microchannel clearly revealed an increment in the number of viable cells over the period of 7 days when compared to the perfusable channel-free 3D construct, thus revealing its cytocompatibility and enhanced diffusion of nutrients and oxygen (Figure 10b). At day 3, the migration of the cells toward the perfusable microchannel was clear, which is nutrient-rich and enables oxygen diffusion. Moreover, at day 5, a well distributed and higher number of viable cells was denoted in the perfusable microchannel-incorporated GelMA construct when compared to the microchannel-free construct. Although a lower cell density was denoted at day 7 on both 3D constructs, it is noticeable that the cells encapsulated on the 3D construct embedding the perfusable microchannel migrated to the middle of the gel, toward the perfusable microchannel. Moreover, at the different time points, it is possible to observe cells in the inner wall of the perfusable microchannel. On the other hand, on the microchannel-free 3D construct, most of the cells were located at the hydrogel edges, where a higher amount of nutrients is available and oxygen exchange prevails. These results reveal the versatility and suitability of the proposed approach to bioengineer complex and larger 3D vascular-mimetic constructs to sustain long-term cell viability, opening new avenues in the regeneration of larger damaged tissues, drug screening, as *in vitro* disease models, or as organ-on-a-chip devices.

## Conclusions

In summary, a novel dynamic HA-functionalized G-quadruplex supramolecular hydrogel was successfully developed at physiological pH by multicomponent self-assembly of G and HA-PBA in the presence of K^+^ ions. The formation, enhanced stability, and selectivity of the G-quadruplex self-assembled hydrogel toward K^+^ ions were unveiled experimentally by the formation of a strong, stable, transparent, and self-supporting hydrogel, and corroborated by MD simulations. The dynamic and reversible nature of the cyclic boronate ester bonds established between G and HA-PBA enabled the preparation of dynamic HA-functionalized G-quadruplex supramolecular hydrogels denoting excellent and fast self-healing behavior, as well as thermo-reversible, injectable, and conductive properties. The formation of self-standing G-quadruplex hydrogel-derived size and shape tunable perfusable microchannels was unveiled by embedding the self-degradable hydrogel, as a sacrificial template, in a photo-cross-linkable GelMA supporting matrix, followed by the immersion of the full construct in PBS/culture medium at 37 °C. By employing a pre-photopolymerization step before the arrangement of the G-quadruplex hydrogel fibers in the desired size and geometry, a versatile and straightforward method could be applied to multiple methacrylated polymeric supporting matrices, even with no associated viscosity. The live/dead cellular assay revealed the cytocompatibility of the developed 3D construct. Moreover, at the different time points, the microchannel-embedded 3D construct showcased a higher number of viable cells and their migration toward the perfusable channel when compared to the microchannel-free 3D bulk construct. Such behavior reveals the enhanced diffusion of oxygen and nutrients ensured by the perfusable microchannels, being essential to sustain long-term cell survival. The self-standing perfusable HA-functionalized G-quadruplex hydrogels hold great promise as a size- and shape-tunable sacrificial biomaterial to be embedded in virtually any type of supporting matrix to bioengineer modular and smart 3D platforms for being used in a plethora of bioapplications.

## Supporting Information Available

HA-functionalized G-quadruplex hydrogel preparation, ^1^H NMR spectra, molecular modeling: additional data and methods, rheological and injectable properties, and optical photographs of perfusable microchannels embedded on different methacrylated supporting baths (PDF)

## Abbreviations

G: guanosine
HA-PBA: 3-aminophenylboronic acid functionalized hyaluronic acid
RT: room temperature
MD: molecular dynamics
^1^H NMR: proton nuclear magnetic resonance spectroscopy
PXRD: powder wide-angle X-ray diffraction
ATR-FTIR: attenuated total reflectance-Fourier transform infrared spectroscopy
ThT: thioflavin T
CD: circular dichroism
STEM: scanning transmission electron microscopy
LVR: linear viscoelastic region
*G*′: storage modulus
*G*″: loss modulus
PDMS: poly(dimethylsiloxane)
GelMA: methacrylated gelatin
LamMA: methacrylated laminarin
DexMA: methacrylated dextran
HAMA: methacrylated hyaluronic acid
