# Supplementary material for: Self-Supporting Hyaluronic Acid-Functionalized G-Quadruplex-Based Perfusable Multicomponent Hydrogels Embedded in Photo-Cross-Linkable Matrices for Bioapplications
Source: Biomacromolecules. 2023 Jun 20;24(7):3380–96. doi: 10.1021/acs.biomac.3c00433 (PMC10336844; doi:10.1021/acs.biomac.3c00433)
Supplement: Supplementary file 1 — bm3c00433_si_001.pdf [file bm3c00433_si_001.pdf]

Supporting Information

Self-Supporting Hyaluronic Acid-Functionalized G-Quadruplex-Based Perfusable Multicomponent Hydrogels Embedded in Photo-Cross-Linkable Matrices for Bioapplications

Vera Sousa, Adérito J. R. Amaral, Edgar J. Castanheira, Igor Marques, João M. M. Rodrigues, Vítor Félix, João Borges, and João F. Mano**

CICECO – Aveiro Institute of Materials, Department of Chemistry, University of Aveiro, Campus Universitário de Santiago, 3810-193 Aveiro, Portugal

* Corresponding authors: joaoborges@ua.pt (J.B.); jmano@ua.pt (J.F.M.)

Table of contents:

1. HA-functionalized G-quadruplex hydrogel preparation ... 3
2. Chemical functionalization of HA ... 4
3. Molecular Modeling: Additional Data & Methods ... 5
   3.1. Extended Discussion ... 5
   3.2. Additional Data ... 6
   3.3. Computational Methods ... 11
      3.3.1. Quantum Calculations ... 12
      3.3.2. Classical force field calculations ... 12
      3.3.3. Boron force field parameters ... 12
      3.3.4. RESP atomic charges of A ... 14
      3.3.5. General MD simulation methods ... 15
4. ^{1}H NMR spectra of HA-functionalized G-quadruplex hydrogel ... 16
5. Rheological properties of the G-quadruplex hydrogels and water content evaluation ... 17
6. Injectable properties ... 18
7. Rheological properties of the GelMA matrix and fabrication of perfusable 3D constructs ... 19
8. References ... 20

1. HA-functionalized G-quadruplex hydrogel preparation

Table S1. Conditions used for the preparation of the hydrogels at all tested concentrations.

HA-PBA	PBA	G	KCl
1.5%	31 mM	42 mM	
2.5%	52 mM	70 mM	250 mM
3.5%	73 mM	98 mM	

2. Chemical functionalization of HA

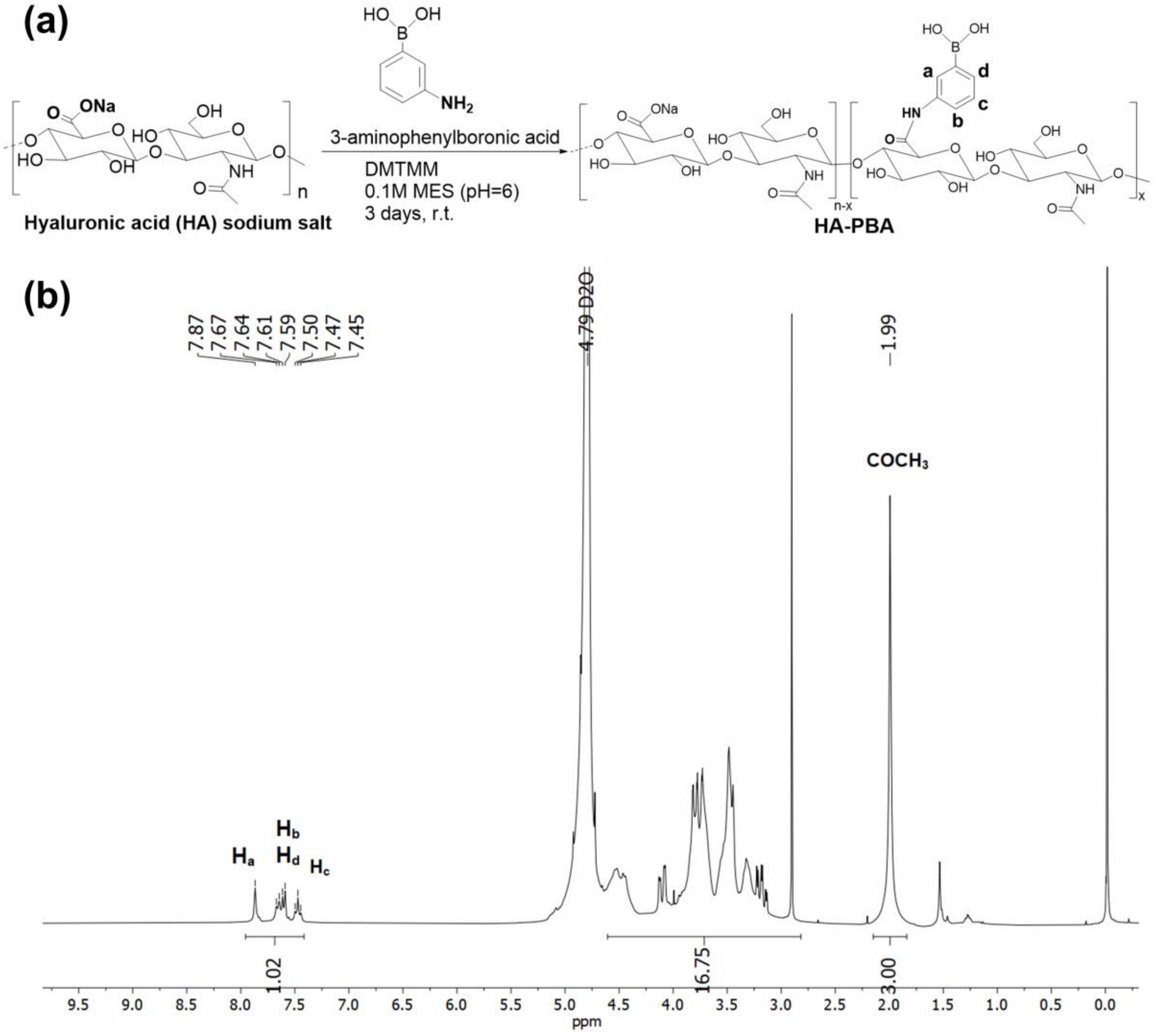

Figure S1. (a) Chemical functionalization of hyaluronic acid with 3-aminophenylboronic acid (HA-PBA). (b) ^{1}H NMR spectrum of HA-PBA at 25 °C in D_2O, 300.13 MHz. The DS was calculated through the multiplication between the ratio of protons responsible for each signal (¾) and the ratio between the integral of the aromatic protons from the conjugated PBA group (δ (ppm) 7.45-7.87, m, 4H) and the HA methyl proton peak (δ (ppm) 1.99, s, 3H).

3. Molecular Modeling: Additional Data & Methods

3.1. Extended Discussion

Following the typical structural assessment of DNA-based G-quadruplexes, the impact of the different cation binders was evaluated with resort to the Root-Mean-Square-Deviations (RMSD) of the G4's, plotted in Figure S3 throughout the MD simulation time. The average values for the concatenated final 10 ns of each MD run are given in Table S2. In line with the dynamical behavior described above, the cation complexes of HA-functionalized G-quadruplex present low average RMSD values, between 1.18 ± 0.29 (Ca^{2+}) and 1.75 ± 0.18 Å (Mg^{2+}), while for Cs^{+} the average RMSD value ascends to 8.16 ± 2.76 Å, being consistent with the structural distortion discussed above. On the other hand, the evaluation of the G-quadruplex's overall Radius of Gyration (ROG, see also Table S2 and Figure S4), due to the bulky HA backbone, yields larger values than in DNA-based G-quadruplexes (*ca*. 17 vs 11 Å).[1] However, the assessment of the G4's ROG leads to values close to 6 Å, being also insufficient to discriminate between the different cations as G-quadruplex binders.

The hydrogen bonding interactions were assessed with cut-offs of 3.0 Å and 120°. The M···O interactions were only considered when the distance between the cation and the oxygen atoms was within a given cut-off. The cut-off values for the different cations were estimated from structures deposited with the CCDC featuring the C=O···M fragment (M = K^{+}, Li^{+}, Na^{+}, Cs^{+}, Mg^{2+} or Ca^{2+}), as follows: 2.3 Å (Li^{+} and Mg^{2+}), 2.6 Å (Na^{+} and Mg^{2+}), 3.0 Å (K^{+}), and 3.5 Å (Cs^{+}). The cut-off value for NH_4^{+} was 3.0 Å, consistent with the typical N···O distances for hydrogen bonds.

3.2. Additional Data

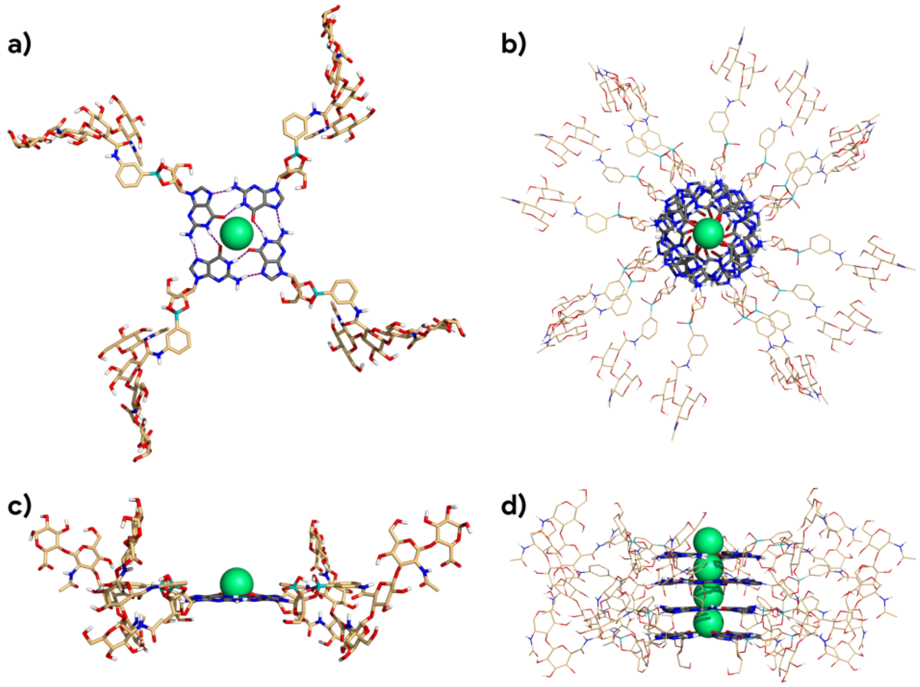

Figure S2. Gas-phase Molecular Mechanics minimised structures of a G4 (a, c) or a model of HA-functionalized G-quadruplex (b, d), respectively coordinated to 1 or 4 K^+ cations (green spheres), in top (a, b) and side views (c, d). Boron is coloured in teal, carbon in light orange or grey (guanine moiety), and the remaining elements follow the typical CPK colouring scheme. For clarity, non-polar H atoms are hidden, while non-guanine atoms of HA-functionalized G-quadruplex are shown as lines. The hydrogen bonds in the G4 are shown as violet dashes.

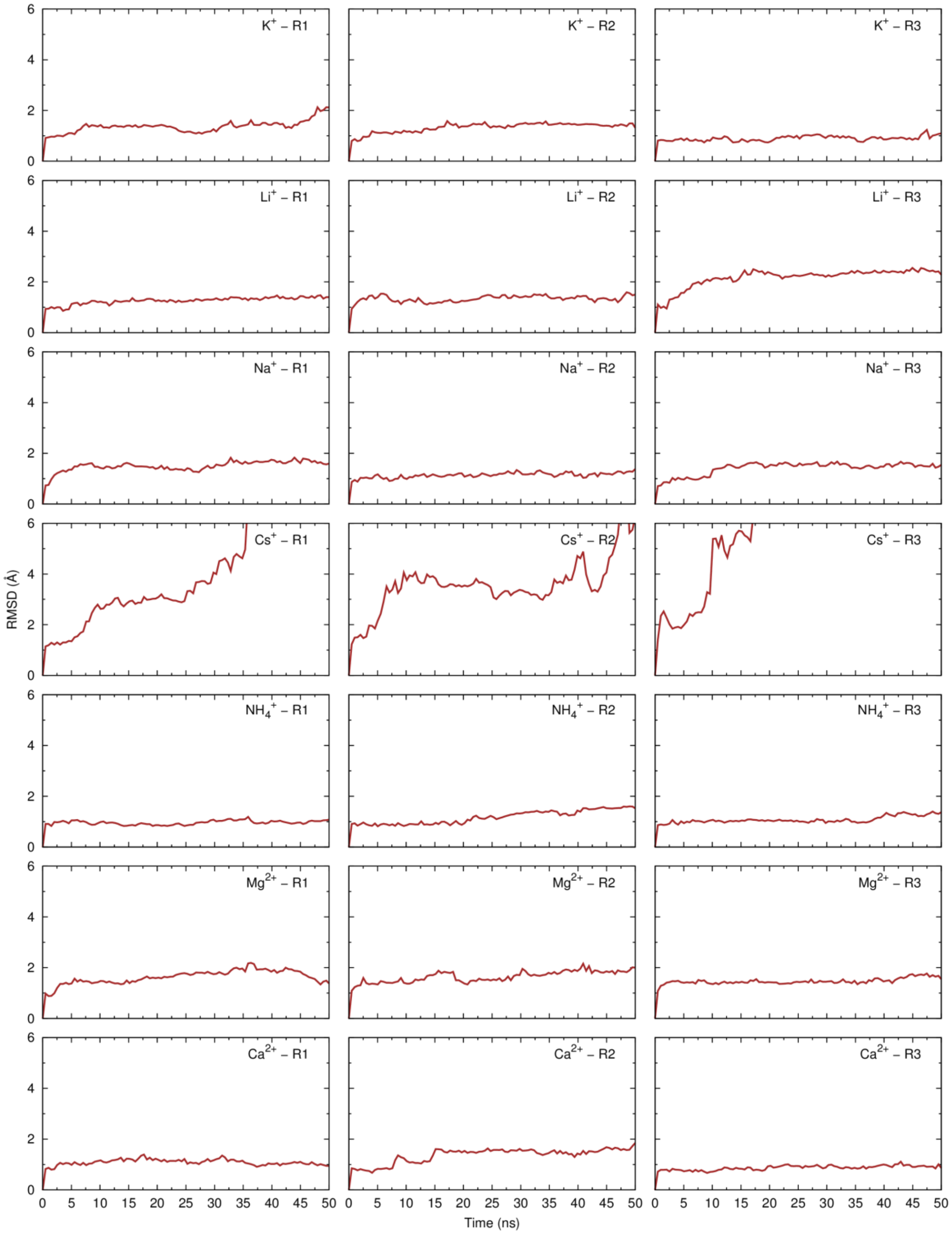

Figure S3. Evolution of the RMSD of the G4 in the HA-functionalized G-quadruplex with different cations. Data were smoothed using Bézier curves.

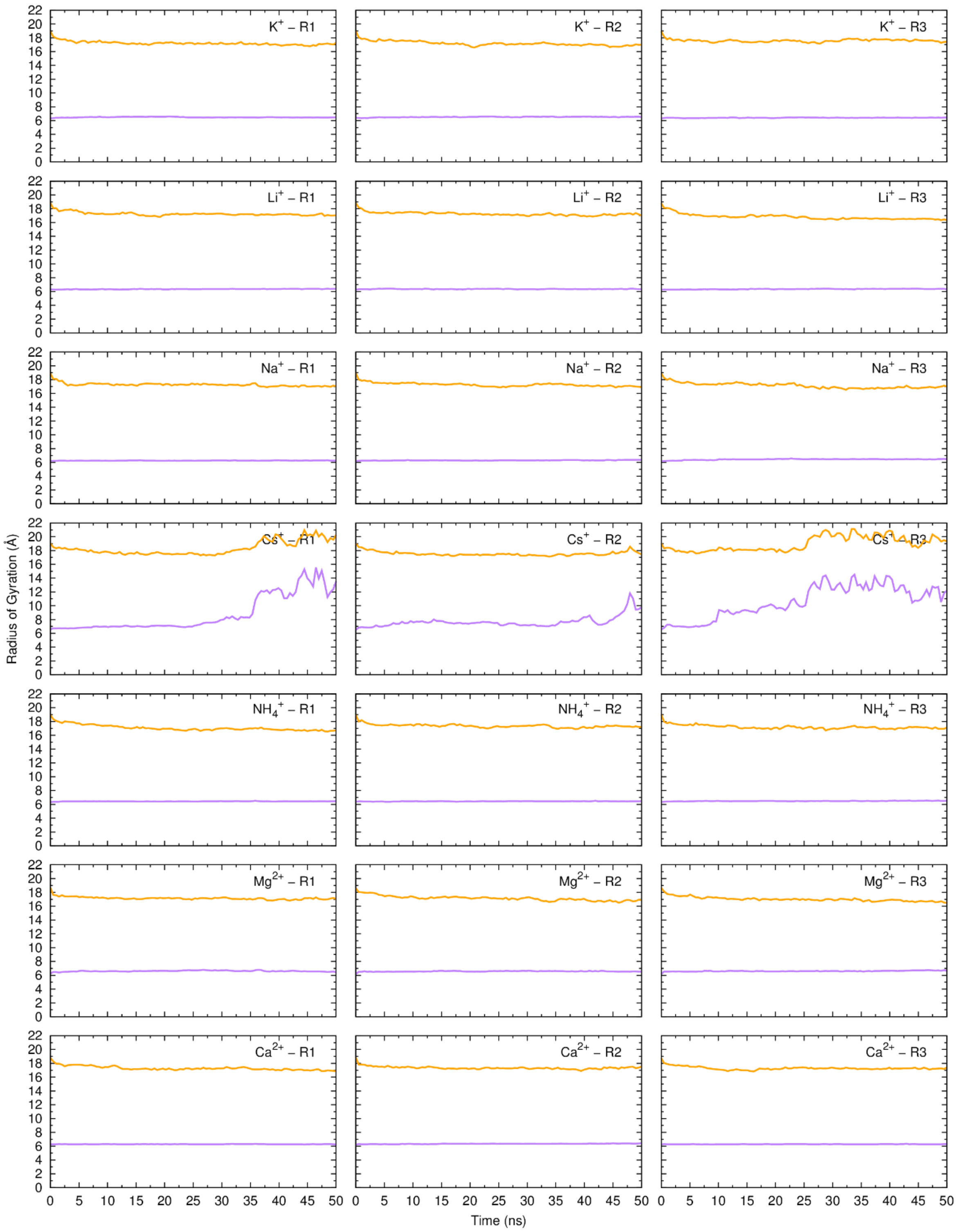

Figure S4. Evolution of the ROG of the G-quadruplex (orange lines) and of the G4 (purple lines) in the MD simulations with different cations. Data were smoothed using Bézier curves.

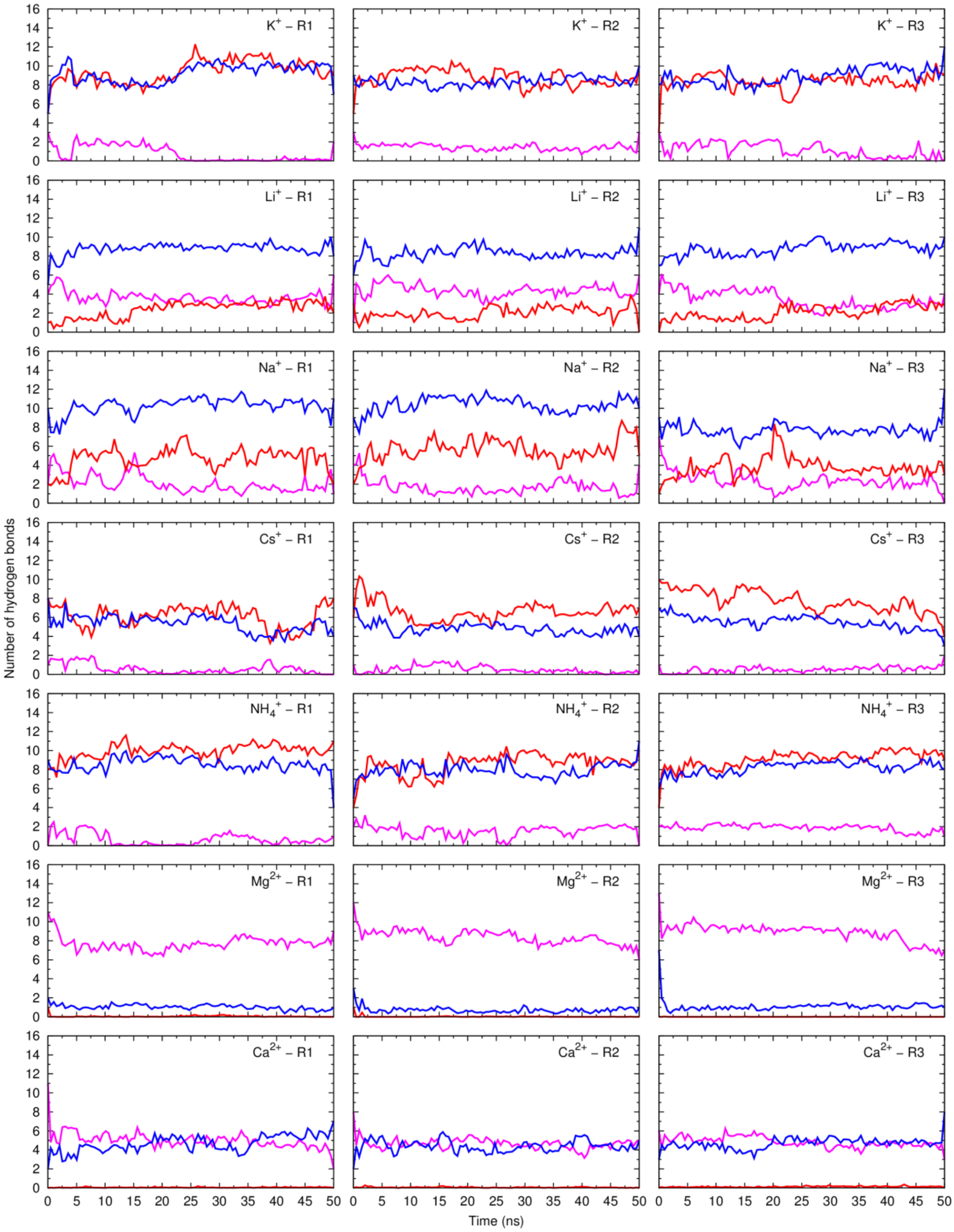

Figure S5. Evolution of intermolecular G4 stabilizing hydrogen bonding interactions, between neighbouring guanine moieties. The N-H···O=C interactions are represented by the red lines, the N-H_2···N ones are drawn in blue and the counting of the N-H···N hydrogen bonds in magenta. Data were smoothed using Bézier curves.

Table S2. Statistics for the G4's RMSD (Å), the G-quadruplex and the G4 ROG (Å) from the concatenated last 10 ns of every MD run for each cation. N = 30000 frames (10000 frames × 3 MD runs).

Cation	G4's RMSD		G-quadruplex's ROG		G4's ROG	
	AVG ± SD	Range	AVG ± SD	Range	AVG ± SD	Range
K^+	1.34 ± 0.33	[0.67 : 2.34]	17.16 ± 0.34	[16.52 : 17.97]	6.46 ± 0.06	[6.30 : 6.70]
Li^+	1.74 ± 0.50	[0.99 : 2.73]	16.91 ± 0.32	[16.22 : 17.59]	6.37 ± 0.04	[6.22 : 6.55]
Na^+	1.47 ± 0.22	[0.86 : 2.04]	17.00 ± 0.14	[16.52 : 17.49]	6.35 ± 0.08	[6.17 : 6.59]
Cs^+	8.16 ± 2.76	[2.99 : 14.42]	18.96 ± 1.11	[17.11 : 21.94]	11.19 ± 2.21	[7.02 : 16.77]
NH_4^+	1.28 ± 0.24	[0.77 : 1.84]	17.01 ± 0.25	[16.38 : 17.62]	6.47 ± 0.05	[6.33 : 6.68]
Mg^{2+}	1.75 ± 0.18	[1.19 : 2.44]	16.85 ± 0.19	[16.37 : 17.51]	6.61 ± 0.08	[6.36 : 6.92]
Ca^{2+}	1.18 ± 0.29	[0.71 : 1.95]	17.19 ± 0.17	[16.66 : 17.67]	6.32 ± 0.06	[6.18 : 6.56]

Table S3. Statistics for the guanine···guanine hydrogen bonds counting throughout the concatenated last 10 ns of every MD run for each cation. N = 30000 frames (10000 frames × 3 MD runs).

Cation	N-H···O=C		$N-H_2\cdots N$		N-H···N	
	AVG ± SD	Range	AVG ± SD	Range	AVG ± SD	Range
K^+	8.9 ± 1.9	[2 : 17]	9.3 ± 2.0	[2 : 16]	0.7 ± 0.9	[0 : 5]
Li^+	2.8 ± 1.5	[0 : 10]	8.7 ± 2.0	[1 : 15]	3.6 ± 1.7	[0 : 11]
Na^+	4.9 ± 2.2	[0 : 14]	9.5 ± 2.3	[0 : 16]	1.7 ± 1.4	[0 : 8]
Cs^+	6.3 ± 1.6	[0 : 12]	4.7 ± 1.4	[0 : 10]	0.4 ± 0.6	[0 : 4]
NH_4^+	9.6 ± 1.6	[2 : 16]	8.3 ± 1.8	[2 : 15]	1.3 ± 1.0	[0 : 5]
Mg^{2+}	0.0 ± 0.1	[0 : 2]	0.8 ± 0.9	[0 : 5]	7.8 ± 1.5	[1 : 13]
Ca^{2+}	0.1 ± 0.3	[0 : 4]	5.0 ± 1.9	[0 : 14]	4.5 ± 1.8	[0 : 12]

Table S4. Statistics for the number of M···O interactions,[a] M···O distances,[b] and M···O=C angles[b] assessed during the concatenated last 10 ns of the three MD runs for each system.

Cation	M···O interactions		M···O (Å)		M···O=C (°)	
	AVG ± SD	Range	AVG ± SD	Range	AVG ± SD	Range
K^+	21.10 ± 1.76	[13.00 : 24.00]	2.73 ± 0.12	[2.36 : 3.00]	131.90 ± 11.03	[85.74 : 179.70]
Li^+	16.02 ± 1.03	[12.00 : 20.00]	2.06 ± 0.09	[1.79 : 2.30]	146.26 ± 13.56	[89.17 : 179.85]
Na^+	16.33 ± 1.42	[10.00 : 22.00]	2.37 ± 0.11	[2.02 : 2.60]	139.77 ± 12.30	[87.88 : 179.78]
Cs^+	12.92 ± 1.68	[4.00 : 17.00]	3.10 ± 0.16	[2.60 : 3.50]	129.43 ± 13.63	[15.84 : 179.90]
NH_4^+	20.83 ± 1.44	[14.00 : 24.00]	2.77 ± 0.10	[2.42 : 3.00]	132.45 ± 12.35	[77.30 : 179.84]
Mg^{2+}	16.00 ± 0.07	[15.00 : 16.00]	2.04 ± 0.06	[1.85 : 2.30]	153.68 ± 10.66	[113.90 : 179.94]
Ca^{2+}	15.84 ± 0.39	[13.00 : 16.00]	2.38 ± 0.07	[2.14 : 2.60]	148.18 ± 10.66	[106.98 : 179.94]

a) M is K^+, Li^+, Na^+, Cs^+, Mg^{2+} or Ca^{2+} or the centre of NH_4^+; and b) For all the frames where the cut-off distance was found.

Table S5. Statistics for the G4···G4 distances (Å) throughout the concatenated last 10 ns of every MD run for each cation.[a]

Cation	G4···G4	
	AVG ± SD	Range
K^+	3.61 ± 0.13	[3.21 : 4.20]
Li^+	3.68 ± 0.14	[3.21 : 4.29]
Na^+	3.54 ± 0.12	[3.10 : 4.21]
Cs^{+b}	6.18 ± 3.25	[2.87 : 20.31]
NH_4^+	3.61 ± 0.15	[3.19 : 4.35]
Mg^{2+}	4.11 ± 0.20	[3.58 : 4.95]
Ca^{2+}	3.58 ± 0.27	[3.15 : 5.02]

a) N = 90000 frames (10000 frames × 3 MD runs × 3 intervals); and b) When considering the best-preserved pair of G4´s the average distance is 3.86 ± 0.56 Å, in the 3.12 to 6.95 Å range.

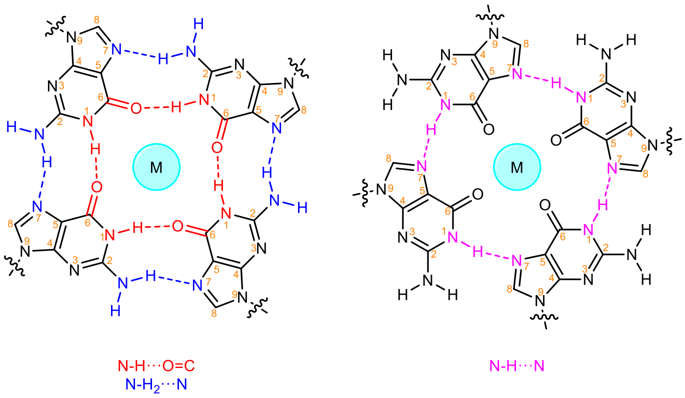

Scheme S1. Putative hydrogen bonding interactions between the guanine moieties of **A,** with the corresponding atom numbering scheme.

3.3. Computational Methods

The Molecular Dynamics (MD) simulations were performed using the G-hyaluronic-based motif **A** (Scheme S2), which was generated from crystal structures deposited with the Protein Data Bank[2] and with the Cambridge Crystallographic Data Centre,[3] by appropriate atomic manipulation. The hyaluronic acid (HA) backbone was taken from 3HYA,[4] whilst the D-glucopyranuronic acid and (3-aminophenyl)-1,3,2-dioxaborole fragments were obtained from WAPWEQ[5] and EHOLOD,[6] respectively, and the G base was acquired from GUANSH10.[7]

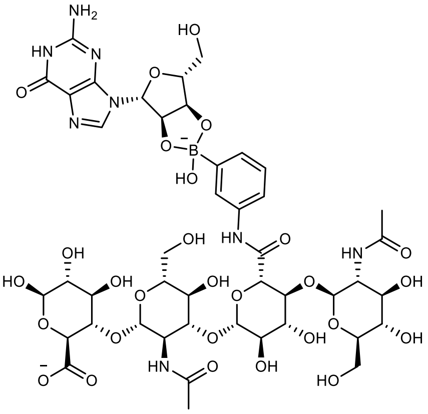

Scheme S2. G-hyaluronic-based motif **A** used in the MD simulations.

The force field parametrisation of the boron bonding terms was carried out using a smaller fragment of **A**, **A***frag*, sketched in Scheme S3.

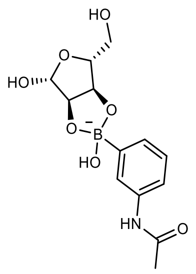

Scheme S3. **A**$_{frag}$ – fragment of **A** used to derive the force field parameters.

3.3.1. Quantum Calculations

The MD simulations were preceded by the development of force field parameters comprising bonding terms for boron and derivatisation of restrained electrostatic potential (RESP) atomic charges, which were performed with Gaussian 16,[8] as detailed below.

3.3.2. Classical force field calculations

The MD simulations were carried out with Amber20,[9] using parameters taken from the second generation of the Generalized Amber Force Field (GAFF2),[10,11] except those involving the boron centre, coupled with RESP atomic charges.[12] The water molecules were described with the TIP3P model,[13] the monoatomic cations were described with a discrete charge of +1 or +2 and van der Waals parameters developed for the TIP3P water model,[14,15] whilst NH_4^+ was described with GAFF2 and standard RESP charges.[10-12] The post-processing of trajectory files to obtain the structural data was performed with *cpptraj*.[16]

3.3.3. Boron force field parameters

The boron bonding terms were derived using the *VFFDT* software[17] and the *paramfit* utility,[18] available within the Amber20 software package. This technical approach has been successfully used in the parametrisation of boronate derivatives.[19,20] The boron van der Waals parameters were directly taken from reference.[21]

The bond length and bond angle terms were estimated for **A**$_{frag}$ using the Seminario method, as implemented in *VFFDT*,[17] from an optimised structure at the B3LYP/6-311++G(2d,2p) level. Their values are listed in Table S, while the corresponding atom types are given in Scheme S4.

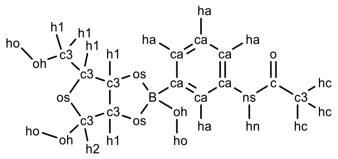

Scheme S4. Gaff2 and custom atom types of **A**$_{frag}$.

The dihedral angle terms centred at the oh-B and ca-B bonds were calculated with *paramfit*, using the following workflow. **A**$_{frag}$ underwent two independent Energy Surface scans through DFT single point energy calculations, with the dihedral angles centred at the oh-B or ca-B bonds being increased by 2° 180 times, resulting in a 360° scan around each bond. Subsequently, the dihedral force field parameters were obtained using the genetic algorithm of *paramfit* in which the MM energies were fitted to the quantum data. The dihedral parameters around the os-B bonds were adapted from the X-os-c3-X parameters available in GAFF2. Unless otherwise specified, all remaining dihedral bond angle parameters as well as the improper terms were adapted from GAFF2, considering that the boron is equivalent to a tetrahedral c3 carbon atom type, as summarised in Table S6.

Table S6. Boron force field parameters used in this work.

**Mass**	**Atomic weight (A_r)**				**Notes**
B	10.81				Taken from ref. [22]
**Bond lengths**	**K_r (kcal mol^{-1} Å^{-2})**	**r_{eq} (Å)**			**Notes**
os-B	124.393	1.497			Determined with *VFFDT*
oh-B	172.808	1.475			Determined with *VFFDT*
ca-B	148.744	1.634			Determined with *VFFDT*
**Bond angles**	**K_θ (kcal mol^{-1} rad^{-2})**	**θ_{eq} (°)**			**Notes**
os-B -os	92.928	103.580			Determined with *VFFDT*
os-B -oh	80.578	109.830			Determined with *VFFDT*
os-B -ca	98.930	110.680			Determined with *VFFDT*
oh-B -ca	100.214	111.930			Determined with *VFFDT*
ho-oh-B	44.696	109.200			Determined with *VFFDT*
ca-ca-B	111.324	121.760			Determined with *VFFDT*
c3-os-B	121.482	108.000			Determined with *VFFDT*
**Dihedral angles**	**Scaling factor**	**$V_n/2$ (kcal mol^{-1})**	**γ (°)**	**periodicity *N***	**Notes**
B -ca-ca-ca	4	14.5000	180.000	2.0	GAFF2 parameters for c3-ca-ca-ca
B -ca-ca-ha	4	14.5000	180.000	2.0	GAFF2 parameters for c3-ca-ca-ha
c3-c3-os-B	1	0.2400	0.000	-3.0	GAFF2 parameters for c3-c3-os-c3
c3-c3-os-B	1	0.1600	0.000	2.0	GAFF2 parameters for c3-c3-os-c3
c3-c3-os-B	1	1.0000	0.000	-2.0	GAFF2 parameters for c3-c3-os-c3
c3-c3-os-B	1	0.0000	0.000	1.0	GAFF2 parameters for c3-c3-os-c3
h1-c3-os-B	1	0.3370	0.000	3.0	GAFF2 parameters for h1-c3-os-c3
ca-B -os-c3	3	1.1500	0.000	3.0	GAFF2 parameters for ca-c3-os-c3
os-B -os-c3	1	0.0000	180.000	-3.0	GAFF2 parameters for os-c3-os-c3
os-B -os-c3	1	1.2400	0.000	-2.0	GAFF2 parameters for os-c3-os-c3
os-B -os-c3	1	0.9700	180.000	1.0	GAFF2 parameters for os-c3-os-c3
oh-B -os-c3	3	1.1500	0.000	3.0	GAFF2 parameters for oh-c3-os-c3
ca-ca-B -os	1	0.0267	0.000	2.0	Determined with *paramfit*
ca-ca-B -oh	1	-0.0546	0.000	2.0	Determined with *paramfit*
os-B -oh-ho	1	0.0386	0.000	3.0	Determined with *paramfit*
ca-B -oh-ho	1	0.3584	0.000	3.0	Determined with *paramfit*
**Improper dihedral**		**K_Φ (kcal mol^{-1})**	**Φ_0 (°)**	**periodicity *N***	**Notes**
B -ca-ca-ca		1.1000	180.000	2.0	GAFF2 parameters for c3-ca-ca-ca
**Van der Waals**	**r (Å)**	**ε (kcal mol^{-1})**			**Notes**
B	1.980	0.0340			Taken from ref. [21]

3.3.4. RESP atomic charges of A

A fragment-based approach was employed for the calculation of the RESP atomic charges, dividing **A** into three components, as sketched in Scheme S5: modified HA (**MHA**), and D-glucuronic acid (**DGA**) and *N*-acetyl-D-glucosamine (**NAG**) as capping groups of central **MHA**. The capping groups' charges were calculated from the prototype sketched in Scheme S6. In agreement with the charge derivatisation philosophy in the force field family,[23] these molecules were optimised at the HF/6-31+G(d) level. Subsequently, the atomic charges were obtained in a two stages RESP charge fitting from the electrostatic potential estimated at the same level of theory. The RESP atomic charges of the **DGA** and **NAG** capping groups were determined considering a global net charge of -1, whilst -1 and 0 charges were respectively attributed to the **DGA** and **NAG** fragments. Having determined the RESP atomic charges for these two capping groups, the **MHA** charges were finally derivatised.

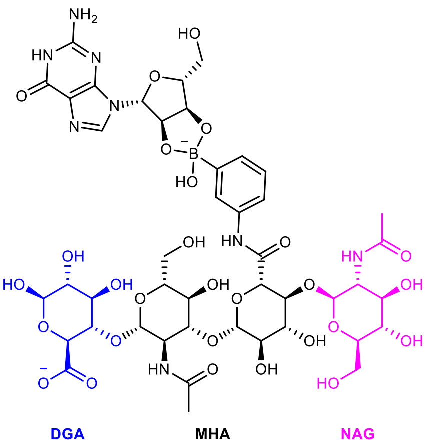

Scheme S5. The three components of **A** used in the atomic charges' derivatization.

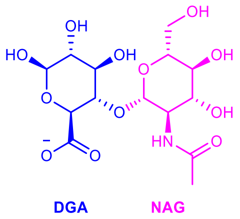

Scheme S6. Capping groups used in the atomic charges' derivatization.

3.3.5. General MD simulation methods

An initial G4 of four **A** molecules surrounding one K^+ cation was built and minimised in the gas phase until the convergence criterion of 0.0001 kcal mol^{-1} was achieved. Afterwards, the structure of the minimised G4 was repeated three times over itself, affording a G-quadruplex assembled by four K^+ coordinated to the guanines' oxygen atoms. This model structure was minimised in the gas phase by MM and used as a starting binding scenario for the subsequent MD simulations, regardless of the cation. Afterwards, this structure was solvated in a truncated octahedron box with 15400 TIP3P model water molecules. For each system, a variable number of cations was added to neutralise its net charge.

Each solvated system was equilibrated under periodic boundary conditions using the following multistage protocol. The system was relaxed by MM minimization of solvent molecules and by keeping all solutes fixed with a positional restraint of 500 kcal mol^{-1} Å^{-2}. The restraint was then removed, and the entire system was allowed to relax. These two minimization stages comprised an initial set of 10000 steepest descent algorithm steps, followed by 10000 steps of conjugated gradient algorithm. The equilibration proceeded with heating up the system to 300 K for 100 ps using an NVT ensemble and a weak positional restraint (10 kcal mol^{-1} Å^{-2}) on the

G-quadruplexes and coordinated cations. Afterwards, maintaining the positional restraints, each system's density was allowed to equilibrate in an NPT ensemble at 1 atm for 1 ns, at the same temperature, followed by an NPT data collection run of 50 ns without any positional restraints. The collection run's trajectory frames were saved every 1 ps. Three independent runs were performed for each system. The CUDA version of the PMEMD executable was used for the simulation of all solvated systems.[24, 25] The bond lengths involving all bonds to hydrogen atoms were constrained with the SHAKE algorithm, allowing the usage of a 2 fs time step.[26] The Particle Mesh Ewald (PME) method was used to treat the long-range electrostatic interactions.[27] The non-bonded van der Waals interactions were truncated with a 10 Å cut-off.

4. ^{1}H NMR spectra of HA-functionalized G-quadruplex hydrogel

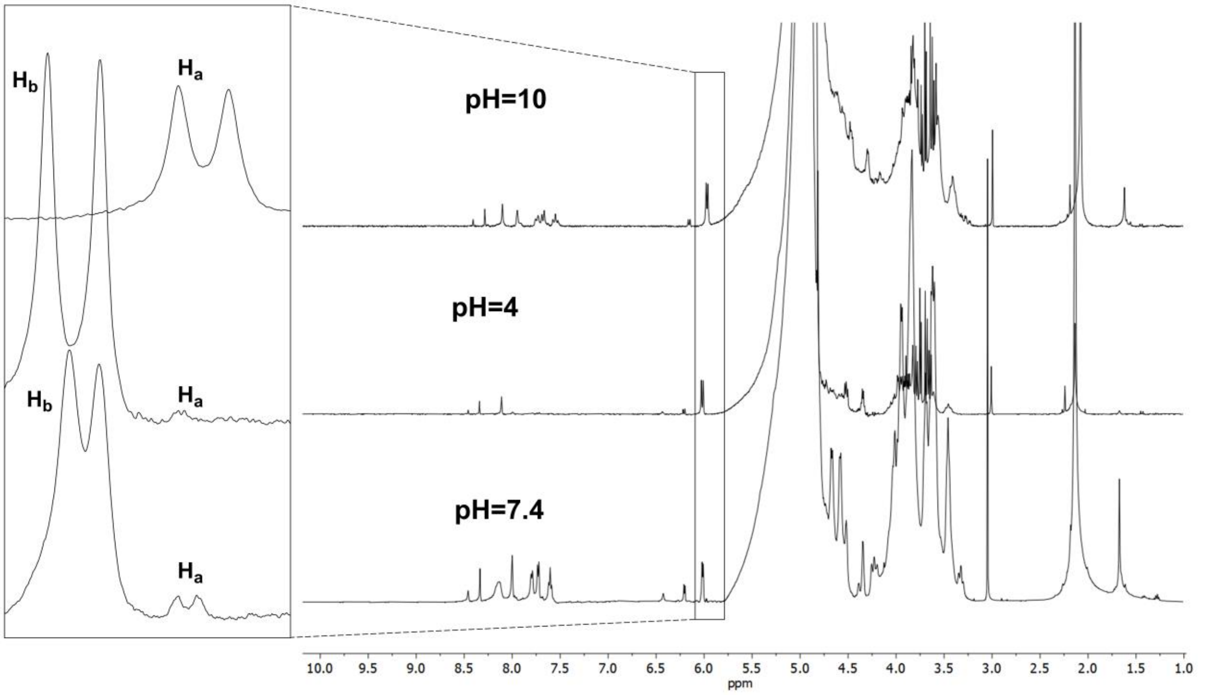

Figure S6. ^{1}H NMR spectra of G-quadruplex hydrogel at pH 4, 7.4, and 10 in D_2O, 300.13 MHz; H_a and H_b refer to reacted and unreacted G, respectively.

5. **Rheological properties of the G-quadruplex hydrogels and water content evaluation**

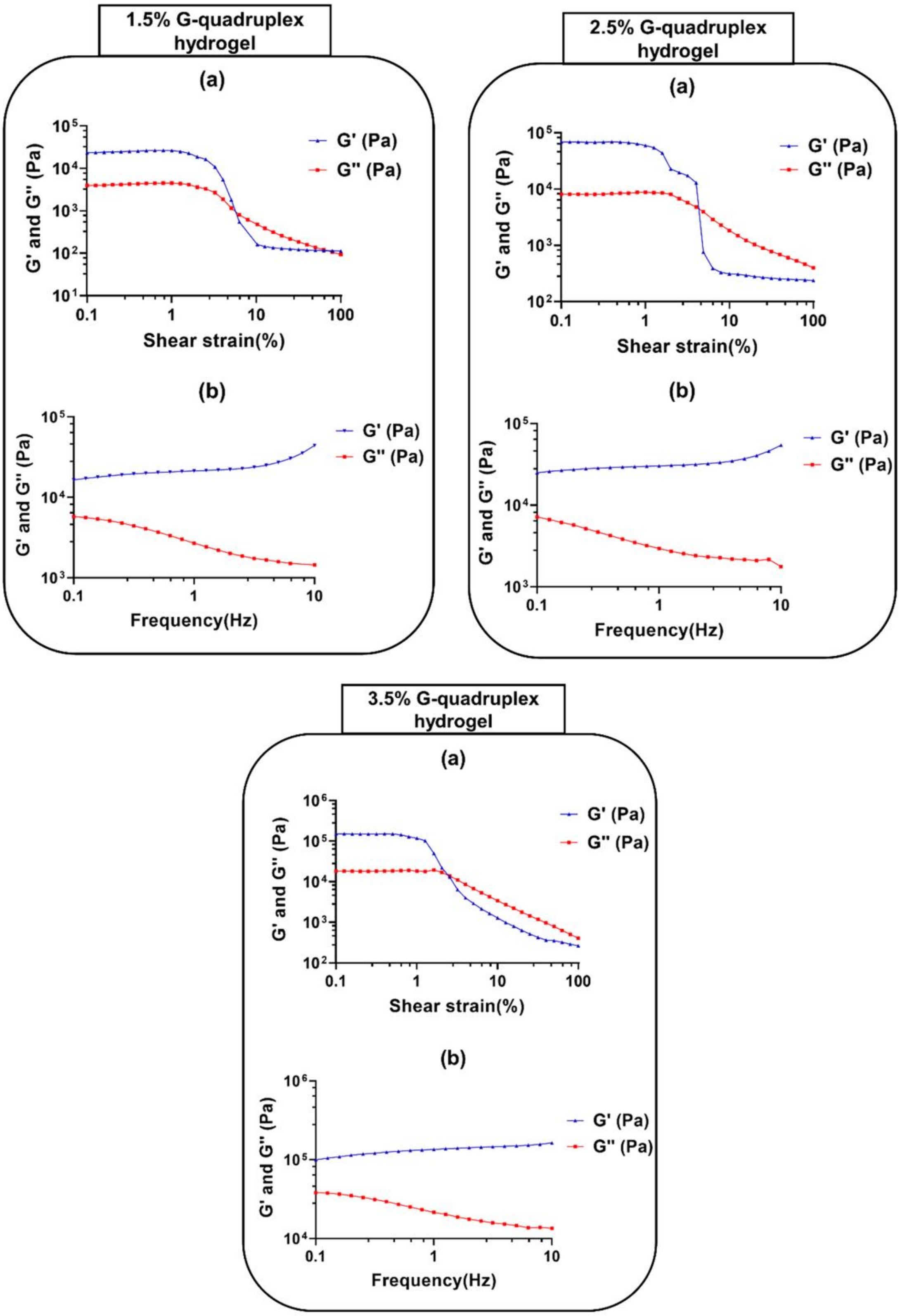

Figure S7. Rheological characterization of 1.5, 2.5 and 3.5% (w/v) HA-functionalized G-quadruplex hydrogels. (a) Strain sweep measurements of hydrogels at fixed 1 Hz. (b) Frequency sweep measurements for all concentrations in the range of 0.1 to 10 Hz (at a constant 0.5% strain).

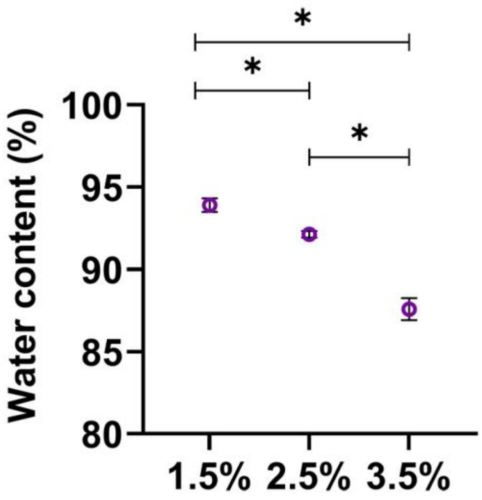

Figure S8. Water content (%) of the hydrogels at all tested concentrations, * $p < 0.05$ ($n = 3$).

6. Injectable properties

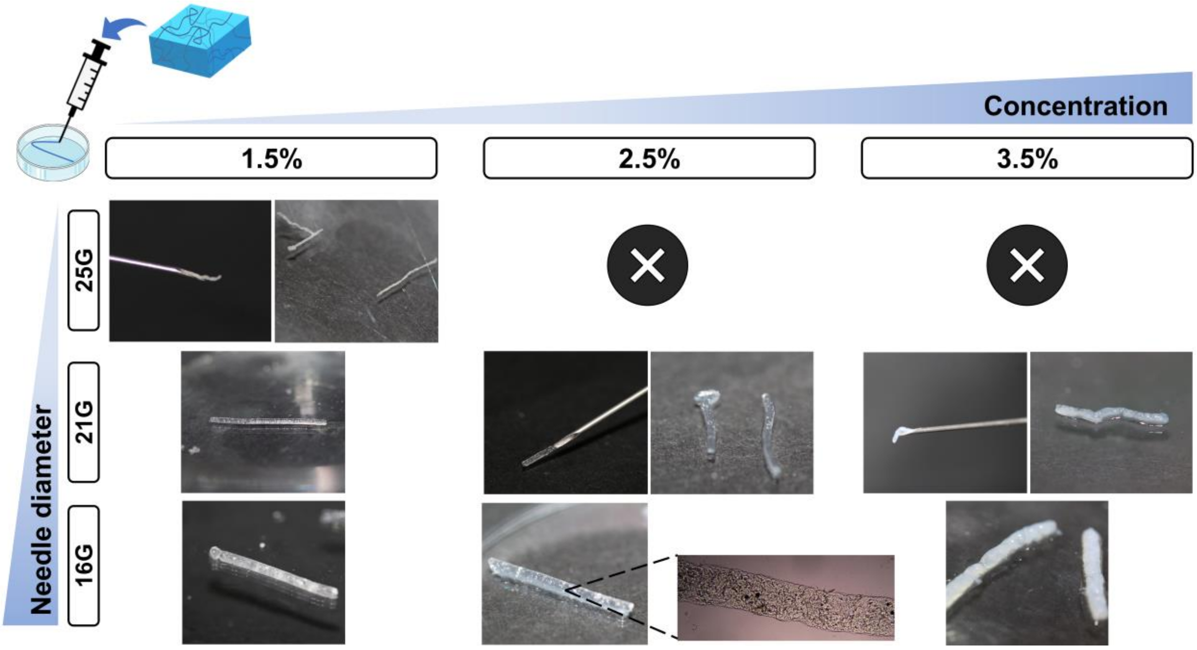

Figure S9. HA-functionalized G-quadruplex hydrogels injection at all concentrations through different needle gauge (25, 21, and 16 G) resulting in well-defined needle-shaped fibres.

7. Rheological properties of the GelMA matrix and fabrication of perfusable 3D constructs

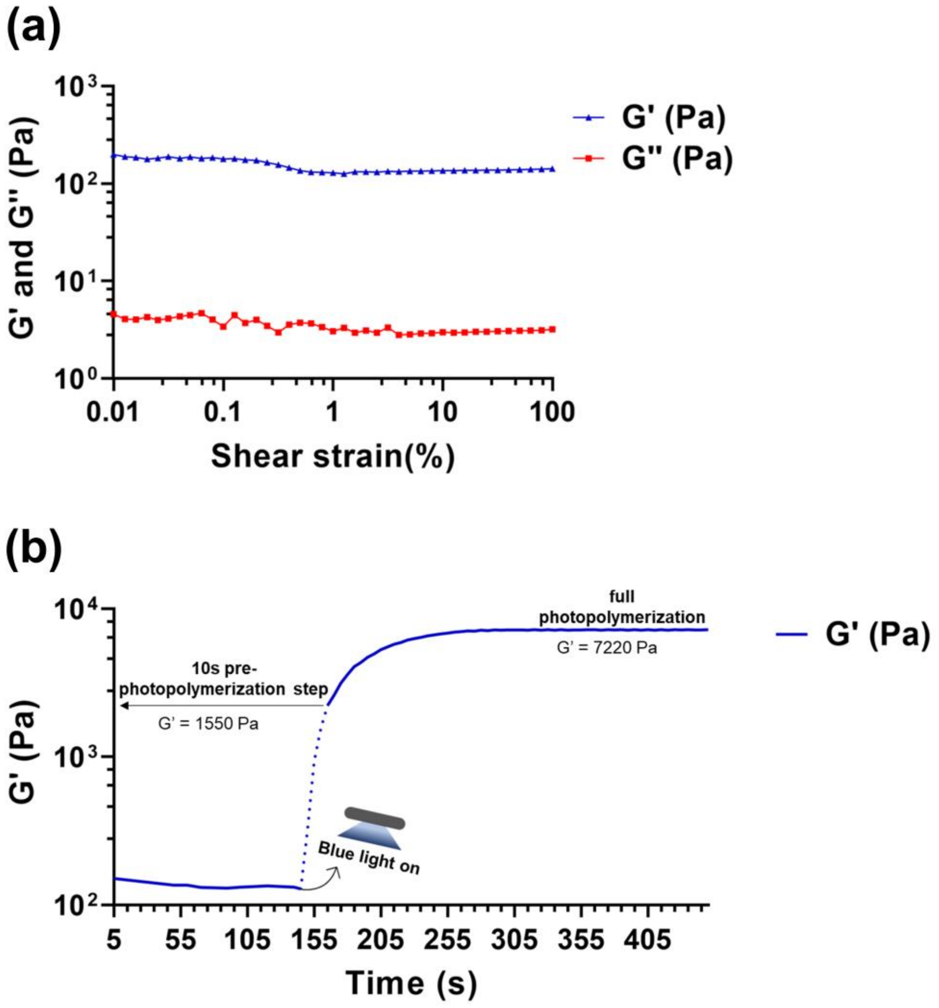

Figure S10. (a) Oscillatory strain sweep measurements of GelMA 10% (w/v) precursor solution at fixed 1 Hz. (b) Gel point determination (time sweep rheology) through irradiation with blue light (λ=385-515 nm) at 25 °C within the LVR (0.03% strain). The light source was switched on at 150 s; in the first 10 s the G′ value increased up to 1550 Pa and continued to rise for the remaining 290 s of the test, achieving a plateau at 7220 Pa.

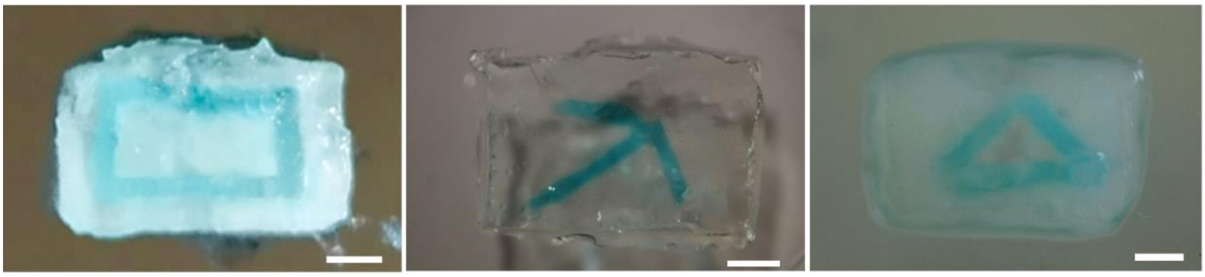

Figure S11. Optical photographs of perfusable microchannels, filled with blue dye, embedded on different methacrylated supporting baths (laminarin (LamHA), hyaluronic acid (HAMA), and dextran (DexMA) at 5% (w/v)). Scale bars: 2.00 mm.

8. References

(1) Carvalho, J.; Santos, T.; Carrilho, R.; Sousa, F.; Salgado, G. F.; Queiroz, J. A.; Cruz, C. Ligand screening to pre-miRNA 149 G-quadruplex investigated by molecular dynamics. *J. Biomol. Struct. Dyn.* **2020**, *38*, 2276-2286.

(2) Berman, H. M.; Westbrook, J.; Feng, Z.; Gilliland, G.; Bhat, T. N.; Weissig, H.; Shindyalov, I. N.; Bourne, P. E. The Protein Data Bank. *Nucleic Acids Res.* **2000**, *28*, 235-242.

(3) Groom, C. R.; Bruno, I. J.; Lightfoot, M. P.; Ward, S. C. The Cambridge Structural Database. *Acta. Crystallogr. B. Struct. Sci. Cryst. Eng. Mater.* **2016**, *72*, 171-179.

(4) Guss, J. M.; Hukins, D. W.; Smith, P. J.; Winter, W. T.; Arnott, S. Hyaluronic acid: molecular conformations and interactions in two sodium salts. *J. Mol. Biol.* **1975**, *95*, 359-384.

(5) Tosin, M.; O'Brien, C.; Fitzpatrick, G. M.; Muller-Bunz, H.; Glass, W. K.; Murphy, P. V. Synthesis and structural analysis of the anilides of glucuronic acid and orientation of the groups on the carbohydrate scaffolding. *J. Org. Chem.* **2005**, *70*, 4096-4106.

(6) Vega, A.; Zarate, M.; Tlahuext, H.; Höpfl, H. Crystal structure of aqua-2-(3-aminophenyl)-1,3,2-dioxaborole dihydrate, $C_{12}H_{12}BNO_3 \cdot 2H_2O$. *Z. Kristallogr. NCS* **2010**, *225*, 676-678.

(7) Thewalt, U.; Bugg, C. E.; Marsh, R. E. The crystal structure of guanosine dihydrate and inosine dihydrate. *Acta. Crystallogr. B. Struct. Sci. Cryst. Eng. Mater.* **1970**, *26*, 1089-1101.

(8) Frisch, M. J.; Trucks, G. W.; Schlegel, H. B.; Scuseria, G. E.; Robb, M. A.; Cheeseman, J. R.; Scalmani, G.; Barone, V.; Petersson, G. A., Nakatsuji, H.; Li, X.; Caricato, M.; Marenich, A. V.; Bloino, J.; Janesko, B. G.; Gomperts, R.; Mennucci, B.; Hratchian, H. P.; Ortiz, J. V.; Izmaylov, A. F.; Sonnenberg, J. L.; Williams-Young, D.; Ding, F.; Lipparini, F.; Egidi, F.; Goings, J.; Peng, B.; Petrone, A.; Henderson, T.; Ranasinghe, D.; Zakrzewski, V. G.; Gao, J.; Rega, N.; Zheng, G.; Liang, W.; Hada, M.; Ehara, M.; Toyota, K.; Fukuda, R.; Hasegawa, J.; Ishida, M.; Nakajima, T.; Honda, Y.; Kitao, O.; Nakai, H.; Vreven, T.; Throssell, K.; Montgomery, J. J. A.; Peralta, J. E.; Ogliaro, F.; Bearpark, M. J.; Heyd, J. J.; Brothers, E. N.; Kudin, K. N.; Staroverov, V. N.; Keith, T. A.; Kobayashi, R.; Normand, J.; Raghavachari, K.; Rendell, A. P.; Burant, J. C.; Iyengar, S. S.; Tomasi, J.; Cossi, M.; Millam, J. M.; Klene, M.; Adamo, C.; Cammi, R.; Ochterski, J. W.; Martin, R. L.; Morokuma, K.; Farkas, O.; Foresman, J. B.and Fox, D. J. *Gaussian 16, Revision C.01*, (2019) Gaussian, Inc., Pittsburgh PA.

(9) Case, D. A.; Belfon, K.; Ben-Shalom, I. Y.; Brozell, S. R.; Cerutti, D. S.; Cheatham, I. T. E.; Cruzeiro, V. W. D.; Darden, T. A.; Duke, R. E.; Giambasu, G.; Gilson, M. K.; Gohlke, H.; Goetz, A. W.; Harris, R.; Izadi, S.; Izmailov, S. A.; Kasavajhala, K.; Kovalenko, A.; Krasny, R.; Kurtzman, T.;

Lee, T. S.; LeGrand, S.; Li, P.; Lin, C.; Liu, J.; Luchko, T.; Luo, R.; Man, V.; Merz, K. M.; Miao, Y.; Mikhailovskii, O.; Monard, G.; Nguyen, H.; Onufriev, A.; Pan, F.; Pantano, S.; Qi, R.; Roe, D. R.; Roitberg, A.; Sagui, C.; Schott-Verdugo, S.; Shen, J.; Simmerling, C. L.; Skrynnikov, N. R.; Smith, J.; Swails, J.; Walker, R. C.; Wang, J.; Wilson, L.; Wolf, R. M.; Wu, X.; Xiong, Y.; Xue, Y.; York, D. M. and Kollman, P. A. *AMBER 2020*, **2020**, University of California, San Francisco.

(10) Wang, J.; Wolf, R. M.; Caldwell, J. W.; Kollman, P. A.; Case, D. A. Development and testing of a general amber force field. *J. Comput. Chem.* **2004**, *25*, 1157-1174.

(11) Wang, J.; Wolf, R. M.; Caldwell, J. W.; Kollman, P. A.; Case, D. A. "Development and testing of a general amber force field"*Journal of Computational Chemistry*(2004) 25(9) 1157–1174. *J. Comput. Chem.* **2005**, *26*, 114-114.

(12) Bayly, C. I.; Cieplak, P.; Cornell, W.; Kollman, P. A. A well-behaved electrostatic potential based method using charge restraints for deriving atomic charges: the RESP model. *J. Phys. Chem.* **2002**, *97*, 10269-10280.

(13) Jorgensen, W. L.; Chandrasekhar, J.; Madura, J. D.; Impey, R. W.; Klein, M. L. Comparison of Simple Potential Functions for Simulating Liquid Water. *J. Chem. Phys.* **1983**, *79*, 926-935.

(14) Li, P.; Roberts, B. P.; Chakravorty, D. K.; Merz, K. M., Jr. Rational Design of Particle Mesh Ewald Compatible Lennard-Jones Parameters for +2 Metal Cations in Explicit Solvent. *J. Chem. Theory. Comput.* **2013**, *9*, 2733-2748.

(15) Li, P.; Song, L. F.; Merz, K. M., Jr. Systematic Parameterization of Monovalent Ions Employing the Nonbonded Model. *J. Chem. Theory. Comput.* **2015**, *11*, 1645-1657.

(16) Roe, D. R.; Cheatham 3rd, T. E. PTRAJ and CPPTRAJ: Software for Processing and Analysis of Molecular Dynamics Trajectory Data. *J. Chem. Theory. Comput.* **2013**, *9*, 3084-3095.

(17) Zheng, S.; Tang, Q.; He, J.; Du, S.; Xu, S.; Wang, C.; Xu, Y.; Lin, F. VFFDT: A New Software for Preparing AMBER Force Field Parameters for Metal-Containing Molecular Systems. *J. Chem. Inf. Model* **2016**, *56*, 811-818.

(18) Betz, R. M.; Walker, R. C. Paramfit: automated optimization of force field parameters for molecular dynamics simulations. *J. Comput. Chem.* **2015**, *36*, 79-87.

(19) Kurt, B.; Temel, H. Parameterization of Boronates Using VFFDT and Paramfit for Molecular Dynamics Simulation. *Molecules* **2020**, *25*, 2196.

(20) Kurt, B.; Temel, H. Development of AMBER parameters for molecular dynamics simulations of boron compounds containing aromatic structure. *Chem. Phys. Lett.* **2021**, *775*, 138656.

(21) Otkidach, D. S.; Pletnev, I. V. Conformational analysis of boron-containing compounds using Gillespie-Kepert version of molecular mechanics. *J. Mol. Struct.* **2001**, *536*, 65-72.

(22) Prohaska, T.; Irrgeher, J.; Benefield, J.; Bohlke, J. K.; Chesson, L. A.; Coplen, T. B.; Ding, T. P.; Dunn, P. J. H.; Groning, M.; Holden, N. E.; et al. Standard atomic weights of the elements 2021 (IUPAC Technical Report). *Pure Appl. Chem.* **2022**, *94*, 573-600.

(23) Kirschner, K. N.; Yongye, A. B.; Tschampel, S. M.; Gonzalez-Outeirino, J.; Daniels, C. R.; Foley, B. L.; Woods, R. J. GLYCAM06: a generalizable biomolecular force field. Carbohydrates. *J. Comput. Chem.* **2008**, *29*, 622-655.

(24) Le Grand, S.; Götz, A. W.; Walker, R. C. SPFP: Speed without compromise—A mixed precision model for GPU accelerated molecular dynamics simulations. *Comput. Phys. Commun.* **2013**, *184*, 374-380.

(25) Salomon-Ferrer, R.; Gotz, A. W.; Poole, D.; Le Grand, S.; Walker, R. C. Routine Microsecond Molecular Dynamics Simulations with AMBER on GPUs. 2. Explicit Solvent Particle Mesh Ewald. *J. Chem. Theory Comput.* **2013**, *9*, 3878-3888.

(26) Ryckaert, J. P.; Ciccotti, G.; Berendsen, H. J. C. Numerical integration of the cartesian equations of motion of a system with constraints: molecular dynamics of n-alkanes. *J. Compu. Phys.* **1977**, *23*, 327-341.

(27) Darden, T.; York, D.; Pedersen, L. Particle Mesh Ewald - an N.Log(N) Method for Ewald Sums in Large Systems. *J. Chem. Phys.* **1993**, *98*, 10089-10092.
